# OMNI: Gas Chromatograph Captures Seven Common PET Radiotracer Analytes in under 5 Minutes

**DOI:** 10.3390/ph16111623

**Published:** 2023-11-17

**Authors:** Camry Vonyae’ A’Keen, Jakub Mroz, Simon Kunta Joseph, Jairo Baquero, Melchor V. Cantorias, Patrick Carberry

**Affiliations:** Department of Radiology, New York University Grossman School of Medicine, 660 First Avenue, Room 240, New York, NY 10016, USAjakub.mroz@nyulangone.org (J.M.); simon.joseph@nyulangone.org (S.K.J.); jairo.baquerobuitrago@nyulangone.org (J.B.); melchor.cantorias@nyulangone.org (M.V.C.)

**Keywords:** gas chromatography, analytes, positron emission tomography, radiotracer, quality control, carbon-11, fluorine-18

## Abstract

A novel gas chromatography method was developed using automatic injections to identify and quantify the amount of residual solvents or analytes in samples of fluorine-18 and carbon-11 radiopharmaceuticals. This approach evaluates seven analytes in less than 5 versus 13 min of acquisition time. The method additionally includes a 3 min bakeout to aid in the removal and carry-over of higher-boiling impurities. Chromatographic parameters such as column temperature, hold time, column pressure, flow rate, and split ratios were adjusted and optimized to analyze radioactive drug samples containing analytes which include methanol, ethanol, acetone, acetonitrile, triethylamine, *N*,*N*-dimethylformamide, and dimethyl sulfoxide. The relative standard deviation for each solvent was determined to be no greater than 1.6%. The method limit of detection (LOD) and limit of quantification (LOQ) were between 0.053 and 0.163 and 0.000 (5.791 × 10^−6^) and 0.520 mg/mL, respectively. This GC technique, using flame ionization detection (FID), was validated and is currently employed for the routine quality control of all approved IND and RDRC PET radiopharmaceuticals at our center.

## 1. Introduction

### 1.1. PET Radiopharmaceuticals

Positron Emission Tomography (PET) is a form of nuclear imaging utilized to observe cellular function within the body. PET employs a radiopharmaceutical agent, commonly referred to as a radiotracer, to visualize and quantify, in real time, the body’s physiological and biochemical processes [1]. The radiotracer is administered to the patient through various routes such as intravenous injection, inhalation, or oral ingestion [2]. Upon entering the body, the radiotracer tends to accumulate in regions exhibiting elevated metabolic or physiologic activity, such as inflamed tissues or cancerous cells. The implementation of concurrent photon emission and detection in PET imaging facilitates a high sensitivity and instantaneous determination of the radiotracer’s concentration and position [3]. The radiotracer comprises an isotope that emits positrons; the annihilation of the ejected positron with electrons in neighboring tissues result in the emission of a pair of gamma rays, which are subsequently captured by detectors on the PET scanner to produce a tridimensional representation of the region under examination [4]. PET is a commonly utilized medical imaging technique in clinical settings to diagnose and monitor various disorders, including but not limited to cancer, Alzheimer’s disease, dementia, schizophrenia, and epilepsy. Moreover, PET, coupled with imaging techniques such as MRI and CT, serves as an invaluable instrument for investigating the intricacies of the brain and comprehending its functionality in both healthy and ill conditions [5].

### 1.2. Gas Chromatography and Flame Ionization Detector

**Figure 1:**
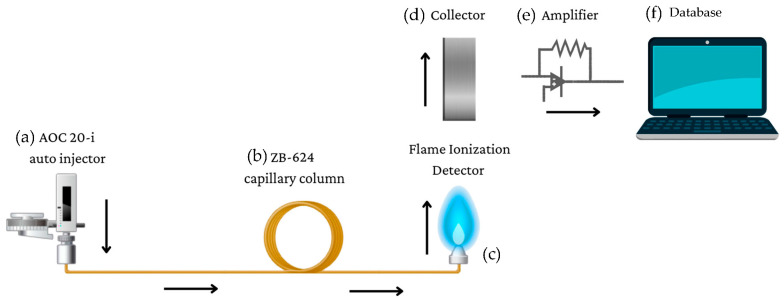
Gas Chromatography Sample Pathway: (**a**) A visual representation by LabSolutions of the sample’s pathway after injection by the AOC 20-i auto injector. (**b**) Pressure adjusted to push mixture through the capillary column and separate the components by chemical composition and boiling point. (**c**) Eluted sample passes through jet, combusting into ions, and (**d**) captured by collector. (**e**) Amplifier that strengthens the current. (**f**) Amplified results displayed via LabSolutions Database.

Gas chromatography (GC) is an effective analytical technique for separating and analyzing volatile and semi-volatile organic compounds [6]. A sample is injected into a GC apparatus, which is outfitted with a separation column, detector, and data system. The sample is vaporized and introduced into the instrument’s injection port, as shown in Figure 1. It then enters a column containing a stationary phase, such as a polymer or porous material, chosen based on the affinity of the molecules being analyzed. As a carrier gas (helium or hydrogen) transports the sample through the column, its components separate based on their attraction to the stationary phase, number of carbon–hydron bonds, and temperature [7]. The separated components then exit the column and enter the detector [8]. A suitable detector, such as a flame ionizing detector (FID), is then used to detect the vaporized compounds [9].

FID is a common GC detector utilized for the determination of analyte quantity, through the detection of ions generated after sample combustion. The process of FID necessitates the controlled combination of hydrogen and column outlet gas at a predetermined ratio, resulting in combustion above the jet tip [10,11]. The jet is subjected to a voltage range of 200–300 V, causing the collection of electrically charged particles by a collector electrode positioned above the flame [12]. An electrical current between the jet and the collector is not created when an inert carrier gas like helium, nitrogen, or hydrogen is present and there is no analyte sample flowing through the column. However, when the carrier gas comprises organic compounds that have attained their individual boiling temperatures, the ions generate an electrical current.

The ion current acquired through the FID is comparatively low, thus strengthened by an amplifier and converted into a corresponding voltage output. After undergoing amplification, the voltage is displayed using a software-processing database (Shimadzu’s LabSolutions), where it is stored. The resulting data are then presented as a peak on a graph quantified by area and height counts, with the x-axis representing time in minutes and the y-axis representing voltage in volts (V) or millivolts (mV). The parts per million (ppm) of the sample can be determined by generating a curve utilizing the area count, which is an arbitrary number, directly corresponding to the amount of analytes found in the sample initially injected [13]. The efficiency of the column and the method produced can be determined by theoretical plates, capacity factor, and the resolution of the produced peaks [14].

### 1.3. Quality Control

GC is commonly used in the nuclear pharmaceutical industry during quality control analyses. The apparatus is typically used to identify and quantify the components present in an injectable sample, such as an extract of a biological matrix or a pharmaceutical formulation. The components under study in a GC analysis, commonly referred to as analytes, are identified and quantified; safety guidelines [15] are followed as passing criteria used for the release of an injectable material to be administered into a patient.

PET imaging techniques frequently require the administration of radiopharmaceuticals, which engage with the physiological processes of the human body. Fluorine-18 and carbon-11 are frequently employed radionuclides for PET imaging. The decay of fluorine-18 occurs through positron (β+) emission and has a half-life of 109.7 min [16], while carbon-11 isotopes have a relatively short half-life of 20.4 min [17,18]. This represents a unique challenge in terms of its production, quality control, and the release of the radiotracer. To limit the loss of activity and ensure the optimal quantity of activity is provided for administration into a patient, it is imperative that these tracers undergo successful synthesis, purification, and quality control testing in accordance with government-mandated regulations in a timely manner. Quality control methods for radiopharmaceuticals must evolve in tandem with the increasing use of novel tracers.

**Figure 2:**
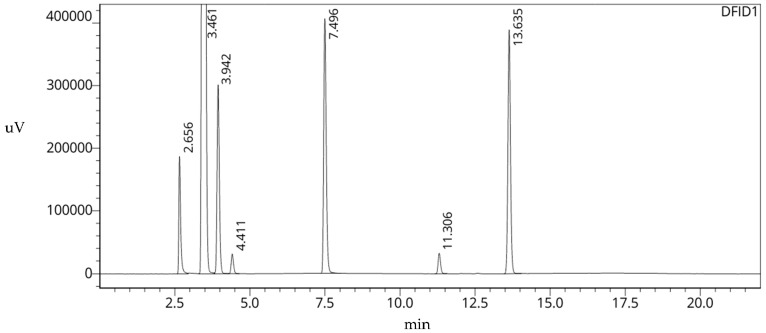
Gas Chromatogram of Original [^11^C]ER176 Method displaying 23 min runtime.

The customary solvents employed at our facility during the production of these radiotracers utilized as either the reaction media or during the purification process include acetonitrile (MeCN), dimethyl sulfoxide (DMSO), *N*,*N*-dimethylformamide (DMF), triethyl amine (Et_3_N), methanol (MeOH), and acetone and ethanol (EtOH) (see Table 1). Due to their chemical nature, it is advisable to monitor the use of these solvents in pharmaceutical-grade products (see Figure 2, Table 2). Various analytical tests are conducted to ensure the safety of radiopharmaceutical tracers for patients. Among these tests, gas chromatography is used to identify and determine the amount of analytes present in a sample before the release of a radiotracer [19].

**Figure 3:**
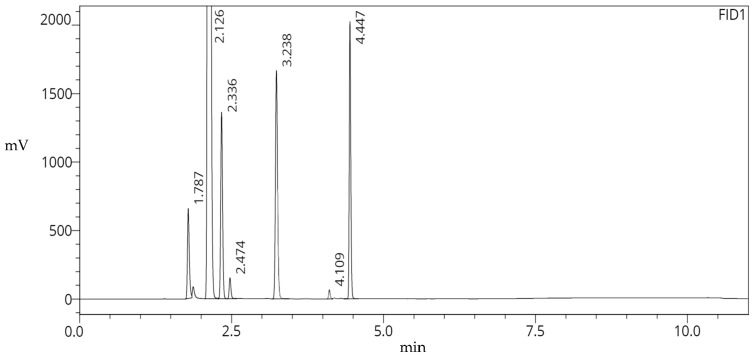
Gas Chromatogram Employing the OMNI Method displaying 11 min runtime.

Our GC quality control test was developed with the specific purpose of expediting quality control procedures for PET radiopharmaceuticals. The objective of this study was to develop a method that encompasses all the solvents used at our site while addressing the issue of effectively isolating volatile organic compounds with diverse levels of polarity and allowable concentrations. To this end, we created an Omniscient Methodology for Novel (pharmaceutical) Injections (OMNI), which meets all the criteria. OMNI is a universal gas chromatography method used at our facility that can analyze up to seven analytes found in various tracers in under a 5 min acquisition time (see Figure 3, Table 3).

This study presents a novel approach that integrates the core principles of gas chromatography, employing commonly utilized materials such as the widely recognized ZB-624 or DB-624 (0.53 mm I.D.) columns in the analysis of pharmaceuticals and volatile organic compounds [20]. This approach integrates retro concepts with an emphasis on the distinct physical and chemical characteristics exhibited by each analyte, i.e., boiling points. Our objective was to develop a single robust and user-centric method that would replace all our existing methods with an efficient, single GC analysis.

## 2. Results

### 2.1. Residual Solvents from Carbon-11 and Fluorine-18 Radiotracer Syntheses

Residual solvents may persist in the context of PET radiotracer production within the field of radiochemistry due to various factors. Radiotracer production conventionally involves a multi-step chemical synthesis process including the synthesis, purification, and extraction of the product. Efforts are undertaken to mitigate the presence of residual solvents to ensure the safety and efficacy of radiotracers, but their presence can be a practical challenge in radiochemistry.

For instance, during the synthesis of [^18^F]-N-(2-(2-fluoroethoxy) benzyl)-N-(4-phenoxypyridin-3-yl) acetamide ([^18^F]FEPPA), methanol is used in the semi-prep mobile phase. During the peak collection of the radiotracer from semi-prep high performance liquid chromatography (HPLC), reagents used for purification (e.g., methanol) are collected in the globe flask of the synthesis module (GE TracerLab FXN Pro by General Electric Healthcare, Waukesha, WI, USA). The globe flask contains 20–50 mL of water, which is used to dilute the collected fraction from purification. The purified product substance undergoes trapping and releasing via a Sep-Pak cartridge that is composed of silica and possesses a pronounced hydrophobic nature. This cartridge is designed to trap the radiotracer, allowing solvent to be rinsed off during the purification process. [21]. The solid phase extraction method does remove all the organic substances used for radiolabeling and purification, and is therefore probable for reagents, such as methanol, to be present. This necessitates checking the quantity of analytes present in the final solution via GC. The permitted daily exposure (PDE) for methanol is 30 mg/day or a concentration of 3000 ppm (see Table 1).

Like [^18^F]FEPPA, N-methyl-11C-2-(4-methylaminophenyl)-6-hydroxybenzothiazole (also known as 11C-6-OH-BTA-1, [¹¹C]Pittsburgh compound-B or [¹¹C]PiB) and (S)-N-[(1 allyl-2-pyrrolidinyl)methyl]-5-(3[^18^F]fluoropropyl)-2,3-dimethoxybenzamide ([^18^F]fallypride) are likely to result in having a residual solvent of triethyl amine (Et_3_N). This occurrence can be attributed to the 0.1 M concentration of Et_3_N in the mobile phase employed for the collection of the final product through HPLC. The PDE for Et_3_N is 50 mg/day or a concentration of 5000 ppm (see Table 1).

Further, the production of (S,S)-[^11^C]methylreboxetine ((S,S)-[^11^C]MRB) requires first dissolving the precursor in DMF. The PDE for DMF is 8.8 mg/day or a concentration of 880 ppm (see Table 1). Similarly, during the production of 11C-(R)-N-sec-butyl-4-(2-chlorophenyl)-N-methylquinazoline-2-carboxamide or [^11^C]ER176, the precursor is initially dissolved in DMSO. The PDE for DMSO is 50 mg/day or a concentration of 5000 ppm. DMSO has a boiling point of 189 °C (see Table 1) and is the last to elute from the column, thus making a typical GC method (see Figure 2, Table 2) for this analyte result in long run times [22].

Acetonitrile is used as a precursor solvent in many radiopharmaceutical synthesis procedures, including those for [^11^C]-meta-hydroxyephedrine ([^11^C]mHED), [^18^F]FEPPA, and [^18^F]fluoro-PEG-folate ([^18^F]PEG-folate). However, this is not the only reason it is tested for during quality control analyses. During the cleaning of the synthesis modules, water, ethanol, acetonitrile, and acetone are used. Without the proper drying of the General Electric synthesis modules for carbon-11 and fluorine-18 compounds, FXC, and FXN, residues will carry over to the final product. The limit for ethanol is 10% of the product solution. The PDE for acetone is 50 mg/day or a concentration of 5000 ppm. The PDE for acetonitrile is 4.1 mg/day or a concentration of 410 ppm.

Methanol, acetonitrile, and DMF are all Class 2 solvents in pharmaceutical products. Ethanol, acetone, Et_3_N, and DMSO are Class 3 solvents which are limited by Good Manufacture Practice (GMP) and quality-based requirements [15]. Despite efforts to purify the radiotracer, the complete removal of all solvents can be challenging. Residual solvents may be retained in the final product due to solubility, reaction kinetics, or incomplete evaporation.

### 2.2. Calibration Curve

**Figure 4:**
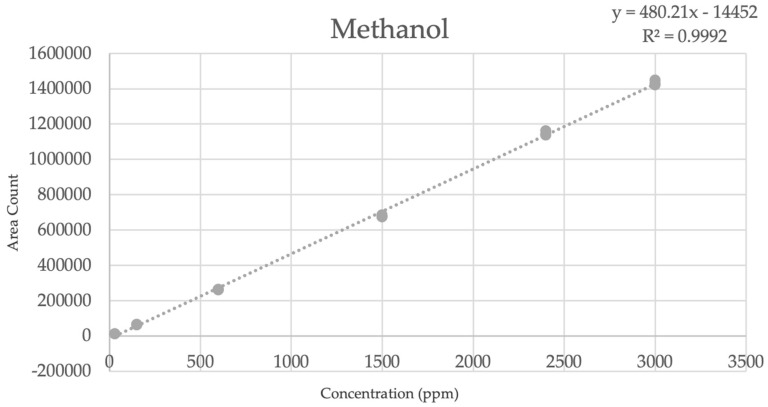
Methanol Curve Plot: Methanol is the first analyte that elutes from the column and has a consistent OMNI retention time of ~1.8 min. This curve has a slope of 480.21, y−intercept of −14,452, and R^2^ of 0.9992.

Utilizing the OMNI standard, a calibration curve was generated on a Shimadzu GC 2030 fitted with an AOC 20i automated injector. The data representing the calibration curve for methanol are shown in Figure 4 and Table 4. For the complete list of all the data for the remaining six analytes found in the OMNI method, please refer to the Supplementary Materials.

The limit of detection (LOD) and limit of quantification (LOQ) were calculated by utilizing the standard deviation and slope of each analyte curve [23]. For this example, the LOD is also regarded as the detection limit (DL) by the ICH guideline [24]. The expression for the DL Equation (1) is given by:

DL = 3.3σ/S, (1)

where σ represents the standard deviation of the response and S is the slope of the calibration curve.

Similarly, the LOQ or quantitation limit (QL) [20] may be stated as:

QL = 10σ/S.(2)

The LOD for the analyte ranged from 0.053 to 0.163 mg/mL with acetone yielding the highest limit. DMF has the highest LOQ, in which with this method can quantify up to 0.520 mg/mL in any give sample. See Table 5 LOD and LOQ results from the calibration curve results.

### 2.3. Validation Experiments

The GC validation experiments were conducted on every radiotracer produced by the NYU Radiochemistry Laboratory to meet all necessary criteria for human injections. Previous protocols include individual standalone methods for analyzing excipients in [^11^C]-ER-176, [^11^C]-MRB, [^11^C]-PiB and [^18^F]-fallypride, and [^18^F]-PEG-folate. Each individual method’s cross-validation is included in the Supplementary Materials. The evaluation of accuracy and precision was conducted during the cross-validation process, using our predefined acceptance criterion based on our original method run on a Shimadzu GC-2014 and approved by Food and Drug Administration (FDA) [24]. Each validation met the criteria within less than 5% RSD difference of each respective method. For a representation of the gas chromatography method’s cross-validation general procedure, please refer to the Supplementary Materials.

## 3. Discussion

The FDA and ICH guidelines outline a process for GC system suitability in radiopharmaceutical preparation and analysis. This process includes instrument performance verification, system suitability testing (SST), standard solution preparation, sample preparation, analysis, and interpretation. The guidelines also emphasize the importance of rigorous quality assurance and control measures throughout the process and are crucial for radiopharmaceutical product safety and efficacy, ensuring the reliability and accuracy of the analytical data generated during manufacturing and testing. The internal SST for our GC consists of a deionized water injection, considered to be a blank injection, demonstrating the absence of volatile substances being injection onto the column. There is a minimum of three consecutive injections of analyte standards consisting of each analyte and corresponding ppm limit, followed by another system blank injection. With a minimum of the necessary five injections, [^11^C]ER-176 SST could take at least 115 min, whereas with OMNI, SST takes 55 min. Refer to the Supplementary Materials for an example of the GC SST performed during the method validation.

The analytes under investigation are subject to the regulatory limits set by the International Council for Harmonisation of Technical Requirements for Pharmaceuticals for Human Use (ICH). Previous methods used in our group and across other radiochemistry facilities had an extended acquisition time (up to 20 min) and did not incorporate all the analytes the OMNI method encompasses [20,25,26]. This prolonged time can be attributed to the chemical properties, boiling point, and elution time of reagents. Due to the short half-life of carbon-11 [18], it is imperative to have an efficient process of gas chromatography without sacrificing analytical sensitivity to determine and quantify analytes. Therefore, it was necessary for us to employ a method that produces a high resolution, reproducibility, and suitable analysis time.

The original methods (a total of six) were produced separately and run on Shimadzu’s GC-2014, with the longest method lasting 23 min. Individual GC methods were used before OMNI, each corresponding to the tracer synthesized during production. These radiotracers are (1) [^11^C]ER-176, (2) [^11^C]MRB, (3) [^11^C]mHED, (4) [^11^C]PiB, (5) [^18^F]FEPPA, and (6) [^18^F]PEG-folate. (7) [^18^F]fallypride used the identical method as [^11^C]PiB, hence why there were only six individual methods. The new OMNI method was developed and optimized on Shimadzu’s GC-2030. The analysis of the seven analytes can be easily integrated after 5 min by utilizing the LabSolutions Snapshot tool. We included a column bakeout process at the end of the comprehensive OMNI method, resulting in a cumulative duration of 11 min. Following the 5 min analysis time, the column temperature was ramped from 210 °C to 250 °C and maintained for a duration of 3 min to facilitate the elution of any remaining volatile residuals, such as dimethyl sulfoxide, that may be present on the column. This additional bake-out time ensured the integrity of the subsequent injections. As a test, a blank sample (deionized water) was injected following this bake-out procedure and no detectable amount of analytes was detected.

Cross-validation data, comprising multiple radiotracers intended for injection into patients undergoing PET scans, can be found in the Supplementary Materials section. The original [^11^C]ER-176 GC method was compared to the new OMNI method using a sample of carbon-11 tracer, specifically from batch: 11C-ER176-101822-01. Based upon Table S9 (original [^11^C]ER-176 GC method) compared to Table S10 (OMNI method), the internal cross-validation demonstrated that they produced nearly identical outcomes. In our study, we observed that the acetone, acetonitrile, and dimethyl sulfoxide exhibited minimal variation among them, whereas the ethanol demonstrated a %RSD difference of 0.02 (please refer to Table S11 in the Supplementary Materials for the results). This study demonstrates the reproducibility and efficiency of the OMNI method using an IND research PET imaging agent at our facility. Another example of the qualification of OMNI used [^18^F]fallypride of batch: 18F-FAL-011323-01 (see Tables S14 and S15 in Supplementary Materials). For our cross-validation method of [^18^F]fallypride (Table S16), the analytes of acetone, acetonitrile, and triethylamine exhibited no significant differences, whereas ethanol showed a slight difference of 0.01%RSD between the two methods. This study validates the application of the OMNI method in a clinical research setting, adhering to the guidelines set forth by the FDA and ICH.

Currently, this method possesses the ability to analyze seven analytes each with a resolution of at least 1.0. The final analyte, dimethyl sulfoxide, elutes at ~4.5 min, allowing for the quality control analyst ample time to conduct additional tests, and for the quality assurance personnel to approve the product for injection; this, in turn, cuts the GC analysis time by a factor of three from several previous methods employed at our center. In the past, these methods were catered to specific tracers; now as the name suggests, our OMNI method is analyte-focused and only requires this method to be performed daily for our system suitability quality control checks. Even so, it is important to note that the current implementation of this method represents only the initial iteration.

## 4. Materials and Methods

### 4.1. Chemicals and Materials

Acetone (Cat. No. 270725), acetonitrile (Cat. No. 34851), ethanol (200 proof, HPLC/spectrophometric grade) (Cat. No. 459828), *N*,*N*-dimethylformamide (Cat. No. 189979), dimethyl sulfoxide (Cat. No. 417939), methanol (Cat. No. 34860), and triethylamine (Cat. No. 471283) were purchased from Sigma Aldrich (St. Louis, MO, USA). Sodium chloride (0.9% USP grade) (Cat. No. NC9054335) by Hospira (Lake Forest, IL, USA). All reagents were used without any further purification. Research-grade compressed air, helium, and hydrogen were purchased from Airgas US, LLC, (Cherryhill, NJ, USA). Bench-top scale with a maximum capacity/readability of 120 g/0.1 mg was manufactured by Mettler Toledo (Columbus, OH, USA). Autosampler 12 × 32 mm borosilicate type I class A (33) glass 200 μL vials with 9 mm PTFE/silicone screw caps were purchased from Shimadzu (Kyoto, Japan) (Part No. 220-91521-03). Sterile empty vials (50 mL) with silver sealed septum (Thermo Fisher Port Washington, New York, NY, USA) were used for creating and storing standards. Syringes (2 mL, Braun Medical Inc., Bethlehem, PA, USA) and sterile needles (20G × 1.5, BD PrecisionGlide™) were used in preparing samples. All pipettes were manufactured by Eppendorf (Hamburg, Germany) and pipette tips by ART™.

### 4.2. OMNI Standard Preparation

In a graduated cylinder, 90 mL of 0.9% sodium chloride was measured. To this solution was added 10 mL of ethanol to provide a 10% ethanol in saline (*v*/*v*) matrix. To a 50 mL volumetric flask was added 10 mL of the 10% ethanol in saline matrix. Using a bench-top scale, the matrix-filled volumetric flask was tared and to this was added acetonitrile (20.5 mg), followed by methanol (150 mg). Additionally, acetone (319 μL), *N*,*N*-dimethylformamide (46.4 μL), dimethyl sulfoxide (227.3 μL), and triethylamine (344.5 μL) were added to the matrix with a calibrated pipettor to fulfill the maximum concentration limits regulated by [15] ICH and used as the standard solution for validating the OMNI method.

### 4.3. Instrumentation

Shimadzu’s Nexus GC-2030 gas chromatograph (Cat. No. C184-0059) equipped with a traditional Phenomenex^®^ Zebron -ZB-624 capillary column (Part No. 7HK-G005-36) was used for this method. The column film thickness was 3.00 μm, with a length of 30.0 m, inner diameter of 0.53 mm, maximum temperature of 260 °C, and minimum temperature of −20 °C. Shimadzu’s GC-2014 gas chromatograph (Cat. No. C184-E014) equipped with a Zebron -ZB-624 column was used for the cross-validation experiments. An autosampler (AOC-20i +s) fitted with a calibrated 10 μL syringe (Part No. S221-34618), needle guide (Part No. S221-44584), plunger holder (Part No. S221-44790), and barrel holder (Part No. S221-44780) was used for all injections into the system. Auto Sampler AOC-20s Plus (Part No. 221-80970-58) was used to hold the 200 μL sampling vials, accelerating the automation of the programed system suitability. Six times of rinsing using a 4 mL vial solvent/waste liquid rack (Part No. S221-32949-01) with deionized water qualified by Milli-Q were performed before and after each injection, with the vacuum and injection speeds set to maximum. Normal injection mode was selected, enabling only the sample to be aspirated with a 1.0 μL injection volume. The temperature and split mode of injection port SPL1 were set to 250 °C. Compressed helium was used as the inert carrier gas with a constant pressure of 27.3 kPa, a total flow rate of 55.5 mL/min, a column flow rate of 4.77 mL/min, a linear velocity of 36.5 cm/s, a purge flow rate of 3.0 mL/min, and a split ratio of 10.0 using a deactivated insert with wool as the split liner (Part No. 227-35007-01) from Shimadzu. Additional parts from Shimadzu includes premium green septa (Part No. S227-35004-01), O-ring for insert (Part No. S227-35005-01), and graphite ferrule for column (Part No. S227-35006-01). LabSolutions LC/GC Software Version 5.99 (Part No. 223-07822-91) was used to develop, display, integrate, and review the results of the OMNI method. LabSolutions, the software used, includes the necessary calculation used to determine resolution and theoretical plates. Maintenance requires regularly replacing the liner, septa, and O-ring weekly and syringe monthly to uphold a low %RSD and achieve satisfactory theoretical plate counts when utilizing OMNI.

**Figure 5:**
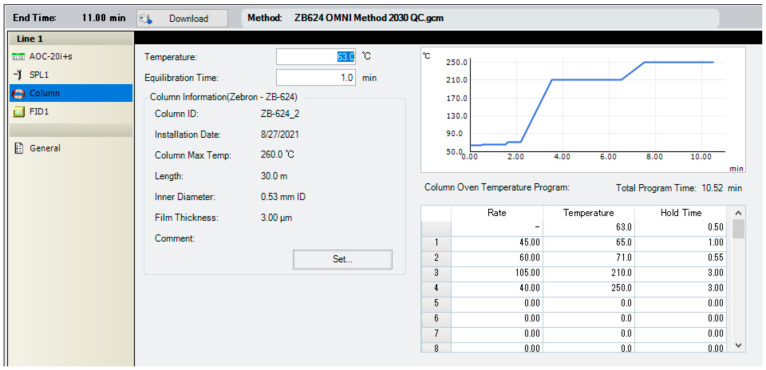
OMNI Column Temperature Ramping Protocol developed in LabSolutions.

The total acquisition time for determining all analytes is under 5 min. The total run-time for the OMNI methods is 11 min. The time from injection to injection can be broken down as such: the initial injection starts at 63 °C for 0.50 min. The column is then ramped to 65 °C for 1.00 min, 71 °C for 0.55 min, and then to 210 °C, with a hold-time of 3.00 min. The method exploits the duration required for the column to reach a temperature range from 71 °C to 210 °C; during which, Et3N, DMF, and DMSO elute off of the column. A bakeout consisting of ramping from 210 °C to 250 °C at a relatively slower rate and hold-time of 3 min is added to the end of the method sequence. This makes the column ramping protocol a total of 8.05 min (Figure 5). The instruments’ built-in cooling system takes roughly 3 min, which brings the full OMNI method to a run-time of 11.00 min. OMNI operates under an automated injection protocol; the autosampler system requires 2 min to rinse and prepare for the subsequent injections. Therefore, the overall injection-to-injection time is 13 min.

## 5. Conclusions

In this study, a unique GC-FID approach was established for detecting seven analytes in flourine-18 and carbon-11 radiopharmaceuticals with injectable limits included in the European Pharmacopoeia. This approach, which incorporates a bakeout to guarantee no carryover from earlier injections, can otherwise give analytical results in less than 5 min acquisition time. The suggested method can quantify methanol, ethanol, acetone, acetonitrile, triethylamine, *N*,*N*-dimethylformamide, and dimethyl sulfoxide in injectable isotonic saline solutions. The OMNI method was cross-validated with prior methods implemented at our facility for the clinical production of radiotracers; the OMNI method demonstrated a good linearity, accuracy, and precision based off of each analytes’ curve data, limit of detection, and limit of quantification (Figure 4), as well as a suitably low RSD% (<1%) across each analyte while dropping the analysis time by more than half. Since the OMNI method was inclusive of all residual solvents in both the fluorine-18- and carbon-11-labeled radiotracers, this procedure only requires one system suitability sequence for the routine quality control analysis of all PET radiopharmaceuticals produced at our facility.

The limitations of this study are that it solely focuses on residual solvents commonly observed at our facility. Tetrahydrofuran (THF) is a common solvent used in synthesis and could be a solvent used in of the purification process. Furthermore, investigating alternative carrier gases, apart from helium, could potentially lead to a reduction in method runtime. Another limitation of our study is the utilization of capillary columns that were already available in our laboratory. It is worth noting that employing a smaller I.D. column could potentially result in improved separation.

Future investigations will delve into various parameters, such as column length, inner diameter, make-up gas flow, and the utilization of hydrogen as a carrier gas [7]. Additionally, we are interested in investigating the volume of injection, as our current practice involves injecting 1 μL. It has been observed that reducing the injection volume to 0.5 μL provides a greater potential for increasing the pressure, thereby resulting in a decreased elution time.

## Figures and Tables

**Table 1:** Common volatile organic compounds found in carbon-11 and flourine-18 radiotracers. * The maximum allowable limit of ethanol concentration at our facility is 10% of the total product volume. ** permitted daily exposure in mg/day.

Analyte	** PDE mg/day	Max ppm	Boling Point °C	ICH Class	Radiotracer
MeOH	30.0	3000	64.7	2	[^18^F]FEPPA
* EtOH	-	-	78.4	3	[^11^C]ER-176, [^11^C]MRB, [^11^C]mHED, [^11^C]PiB, [^18^F]FEPPA [^18^F]PEG-folate, [^18^F]fallypride
Acetone	50.0	5000	56.1	3	[^11^C]ER-176, [^11^C]MRB, [^11^C]mHED, [^11^C]PiB, [^18^F]FEPPA [^18^F]PEG-folate, [^18^F]fallypride
MeCN	4.1	410	82.0	2	[^11^C]ER-176, [^11^C]MRB, [^11^C]mHED, [^11^C]PiB, [^18^F]FEPPA [^18^F]PEG-folate, [^18^F]fallypride
Et_3_N	50.0	5000	89.3	3	[^11^C]PiB, [^18^F]fallypride
DMF	8.8	880	153.0	2	[^11^C]MRB
DMSO	50.0	5000	189.0	3	[^11^C]ER-176

**Table 2:** Gas Chromatogram of Original [^11^C]ER176 Method Data. Theoretical Plates and Resolution calculations are obtained via LabSolutions post-run database, implementing USP guidelines.

Peak No.	Analyte	t_R_	Area Counts	No. of Theoretical Plates (USP)	Capacity Factor (k’)	Resolution (USP)
1	MeOH	2.656	779,157	10,898	--	--
2	EtOH	3.461	38,475,947	11,352	0.303	6.942
3	Acetone	3.942	1,591,392	13,095	0.484	3.595
4	MeCN	4.411	162,192	16,464	0.661	3.412
5	Et_3_N	7.496	2,354,530	39,802	1.822	21.438
6	DMF	11.306	178,246	97,034	3.256	25.787
7	DMSO	13.635	2,284,000	125,475	4.133	15.567

**Table 3:** Data of Gas Chromatogram Employing the OMNI Method. Theoretical Plates and Resolution calculations are obtained via LabSolutions post-run database, implementing USP guidelines.

Peak No.	Analyte	t_R_	Area Counts	No. of Theoretical Plates (USP)	Capacity Factor (k’)	Resolution (USP)
1	MeOH	1.787	1,371,438	18,888	--	--
2	EtOH	2.126	74,289,866	18,323	0.190	5.915
3	Acetone	2.336	3,143,945	28,063	0.302	3.390
4	MeCN	2.474	356,109	37,694	0.378	2.554
5	Et_3_N	3.238	4,537,634	45,124	0.798	13.490
6	DMF	4.109	348,842	138,302	1.291	16.843
7	DMSO	4.447	3,502,774	150,775	1.486	7.749

**Table 4:** Methanol curve data.

Level	ppm	Area Count	Average Area	%RSD	t_R_
1	30 30 30	12,924 12,935 13,012	12,957	0.37	1.841 1.841 1.841
2	150 150 150	65,946 63,773 65,116	64,945	1.69	1.840 1.841 1.841
3	600 600 600	264,117 263,127 262,302	263,182	0.35	1.841 1.841 1.841
4	1500 1500 1500	676,487 685,536 675,753	679,259	0.80	1.842 1.842 1.842
5	2400 2400 2400	1,136,693 1,143,912 1,160,962	1,147,189	1.09	1.843 1.843 1.843
6	3000 3000 3000	1,448,227 1,422,393 1,430,712	1,433,777	0.92	1.843 1.844 1.843

**Table 5:** Limit of Detection and Limit of Quantification in mg/mL.

Analyte	LOD	LOQ
MeOH	0.053	0.094
EtOH	0.056	5.791 × 10^−6^
Acetone	0.067	0.088
MeCN	0.163	0.156
Et_3_N	0.080	0.081
DMF	0.117	0.520
DMSO	0.110	0.137

## Data Availability

The data presented in this study are available in Supplementary Materials.

## Supplementary Materials

The following supporting information can be downloaded at: https://www.mdpi.com/article/10.3390/ph16111623/s1, Figure S1: AOC-20i Parameters Set for OMNI in LabSolutions, Figure S2: Split Injector Parameters Set for OMNI in LabSolutions, Table S1: Ethanol Concentration Curve Data, Figure S3. Ethanol Concentration Curve Graph Table S2: Acetone Concentration Curve Data, Figure S4. Acetone Concentration Curve Graph, Table S3: Acetonitrile Concentration Curve Data, Figure S5. Acetonitrile Concentration Curve Graph, Table S4: Triethylamine Concentration Curve Data, Figure S6. Triethylamine Concentration Curve Graph, Table S5: *N*,*N*-dimethylformamide Concentration Curve Data, Figure S7. *N*,*N*-dimethylformamide Concentration Curve Graph, Table S6: Dimethyl Sulfoxide Concentration Curve Data, Figure S8: Dimethyl Sulfoxide Concentration Curve Graph, Figure S9: Chromatograph for Level 1 Injection 1, Figure S10: Chromatograph for Level 1 Injection 2, Figure S11. Chromatograph for Level 1 Injection 3, Figure S12: Chromatograph for Level 2 Injection 1, Figure S13: Chromatograph for Level 2 Injection 2, Figure S14: Chromatograph for Level 2 Injection 3, Figure S15: Chromatograph for Level 3 Injection 1, Figure S16: Chromatograph for Level 3 Injection 2, Figure S17: Chromatograph for Level 3 Injection 3, Figure S18: Chromatograph for Level 4 Injection 1, Figure S19. Chromatograph for Level 4 Injection 2, Figure S20: Chromatograph for Level 4 Injection 3, Figure S21: Chromatograph for Level 5 Injection 1, Figure S22: Chromatograph for Level 5 Injection 2, Figure S23: Chromatograph for Level 5 Injection 3, Figure S24: Chromatograph for Level 6 Injection 1, Figure S25: Chromatograph for Level 6 Injection 2, Figure S26: Chromatograph for Level 6 Injection 3, Figure S27: Chromatograph for Blank-01 Water Injection After OMNI, Figure S28: Chromatograph for Blank-02 Water Injection After OMNI Table S7: System suitability performed on GC-2014 with original gas chromatography method for [^11^C]ER-176 using standard containing only acetone, ethanol, acetonitrile and DMSO. Table S8: System suitability performed on GC-2030 with new met method using standard containing only acetone, ethanol, acetonitrile and DMSO Table S9: Results from injectable [^11^C]ER-176 Sample using original ZB624 ER-176 method QC Method. Table S10: Results from injectable [^11^C]ER-176 Sample using OMNI Method 2030 QC, Table S11: Method comparison and cross-validation results for [^11^C]ER-176, Table S12: System suitability performed on GC-2014 with original gas chromatography method for [^11^C]PiB and [^18^F]fallypride using standard containing only acetone, ethanol, acetonitrile and triethyl amine, Table S13: System suitability performed on GC-2030 with new met method using standard containing only acetone, ethanol, acetonitrile and triethyl amine, Table S14: Results from injectable [^18^F]fallypride Sample using original ZB624 GC QC Method, Table S15: Results from injectable [^18^F]fallypride Sample using OMNI Method 2030 QC, Table S16: Method comparison and cross-validation results for [^18^F]fallypride, Table S17: System suitability performed on GC-2014 with original gas chromatography method for [^18^F]FEPPA using standard containing only methanol, ethanol, and acetonitrile, Table S18: System suitability performed on GC-2030 with new met method using standard containing only acetone, ethanol, acetonitrile and triethyl amine, Table S19: Results from injectable [^18^F]FEPPA Sample using original ZB624 FEPPA QC Method, Table S20: Results from injectable [^18^F]FEPPA Sample using OMNI Method 2030 QC, Table S21: Method comparison and cross-validation results for [^18^F]FEPPA.
