# Supplementary material for: OMNI: Gas Chromatograph Captures Seven Common PET Radiotracer Analytes in under 5 Minutes
Source: Pharmaceuticals (Basel). 2023 Nov 17;16(11):1623. doi: 10.3390/ph16111623 (PMC10675356; doi:10.3390/ph16111623)
Supplement: Supplementary file 1 [file pharmaceuticals-16-01623-s001.zip › pharmaceuticals-2657470-supplementary.pdf]

pharmaceuticals

Supplementary Materials

OMNI: Gas Chromatograph Captures 7 Common PET Radiotracer Analytes in Under 5 Minutes

Camry Vonyae' A'Keen[1], Jakub Mroz[1], Simon Kunta Joseph[1], Jairo Baquero[1] Melchor Cantorias[1] and Patrick Carberry[1,*]

[1] Department of Radiology, New York University Grossman School of Medicine, 660 First Avenue, Room 240, New York, NY 10016, USA
* Correspondence: patrick.carberry@nyulangone.org; Tel.: 1-646-501-9607

Table of Contents

OMNI Method Parameters via LabSolutions

Figure S1. AOC-20i Parameters Set for OMNI in LabSolutions S4

Figure S2. Split Injector Parameters Set for OMNI in LabSolutions S4

Analyte Curve Plot and Data

Table S1: Ethanol Concentration Curve Data S5

Figure S3. Ethanol Concentration Curve Graph S5

Table S2: Acetone Concentration Curve Data S6

Figure S4. Acetone Concentration Curve Graph S6

Table S3: Acetonitrile Concentration Curve Data S7

Figure S5. Acetonitrile Concentration Curve Graph S7

Table S4: Triethylamine Concentration Curve Data S8

Figure S6. Triethylamine Concentration Curve Graph S8

Table S5 : *N,N*-dimethylformamide Concentration Curve Data S9

Figure S7. *N,N*-dimethylformamide Concentration Curve Graph S9

Table S6: Dimethyl Sulfoxide Concentration Curve Data S10

Figure S8: Dimethyl Sulfoxide Concentration Curve Graph S10

Analysis Report for Concentration Curve

Figure S9: Chromatograph for Level 1 Injection 1 ....S11

Figure S10: Chromatograph for Level 1 Injection 2 ....S12

Figure S11. Chromatograph for Level 1 Injection 3 ....S13

Figure S12: Chromatograph for Level 2 Injection 1 ....S14

Figure S13: Chromatograph for Level 2 Injection 2 ....S15

Figure S14: Chromatograph for Level 2 Injection 3 ....S16

Figure S15: Chromatograph for Level 3 Injection 1 ....S17

Figure S16: Chromatograph for Level 3 Injection 2 ....S18

Figure S17: Chromatograph for Level 3 Injection 3 ....S19

Figure S18: Chromatograph for Level 4 Injection 1 ....S20

Figure S19. Chromatograph for Level 4 Injection 2 ....S21

Figure S20: Chromatograph for Level 4 Injection 3 ....S22

Figure S21: Chromatograph for Level 5 Injection 1 ....S23

Figure S22. Chromatograph for Level 5 Injection 2 ....S24

Figure S23. Chromatograph for Level 5 Injection 3 ....S25

Figure S24: Chromatograph for Level 6 Injection 1 ....S26

Figure S25. Chromatograph for Level 6 Injection 2 ....S27

Figure S26: Chromatograph for Level 6 Injection 3 ....S28

Analysis Report for OMNI Blank Injections

Figure S27: Chromatograph for Blank-01 Water Injection After OMNI S29

Figure S28: Chromatograph for Blank-02 Water Injection After OMNI S30

Cross-Validation Results for [11C]ER-176 and OMNI Method

Table S7. System suitability performed on GC-2014 with original gas chromatography method for [11C]ER-176 using standard containing only acetone, ethanol, acetonitrile and DMSO..S31

Table S8: System suitability performed on GC-2030 with new met method using standard containing only acetone, ethanol, acetonitrile and DMSO..S31

Table S9. Results from injectable [11C]ER-176 Sample using original ZB624 ER-176 QC Method..........................S31

Table S10: Results from injectable [11C]ER-176 Sample using OMNI Method 2030 QC S31

Table S11: Method comparison and cross-validation results for [11C]ER-176 ....S32

Cross-Validation Results for [11C]PiB and [18F]fallypride and OMNI Method

Table S12: System suitability performed on GC-2014 with original gas chromatography method for [11C]PiB and [18F]fallypride using standard containing only acetone, ethanol, acetonitrile and triethyl amine S32

Table S13: System suitability performed on GC-2030 with new met method using standard containing only acetone, ethanol, acetonitrile and triethyl amine S32

Table S14: Results from injectable [18F]fallypride Sample using original ZB624 GC QC Method S33

Table S15: Results from injectable [18F]fallypride Sample using OMNI Method 2030 QC S33

Table S16: Method comparison and cross-validation results for [18F]fallypride, ……………………………………………... S33

Cross-Validation Results for [18F]FEPPA and OMNI Method

Table S17: System suitability performed on GC-2014 with original gas chromatography method for [18F]FEPPA using standard containing only methanol, ethanol, and acetonitrile ……………………………………………………………………………… S34

Table S18: System suitability performed on GC-2030 with new met method using standard containing only acetone, ethanol, acetonitrile and triethyl amine ….……………………………………………………………………………...………………. S34

Table S19: Results from injectable [18F]FEPPA Sample using original ZB624 FEPPA QC Method ……………………………… S34

Table S20: Results from injectable [18F]FEPPA Sample using OMNI Method 2030 QC ….………………………………………. S34

Table S21: Method comparison and cross-validation results for [18F]FEPPA ……………………………………………………... S35

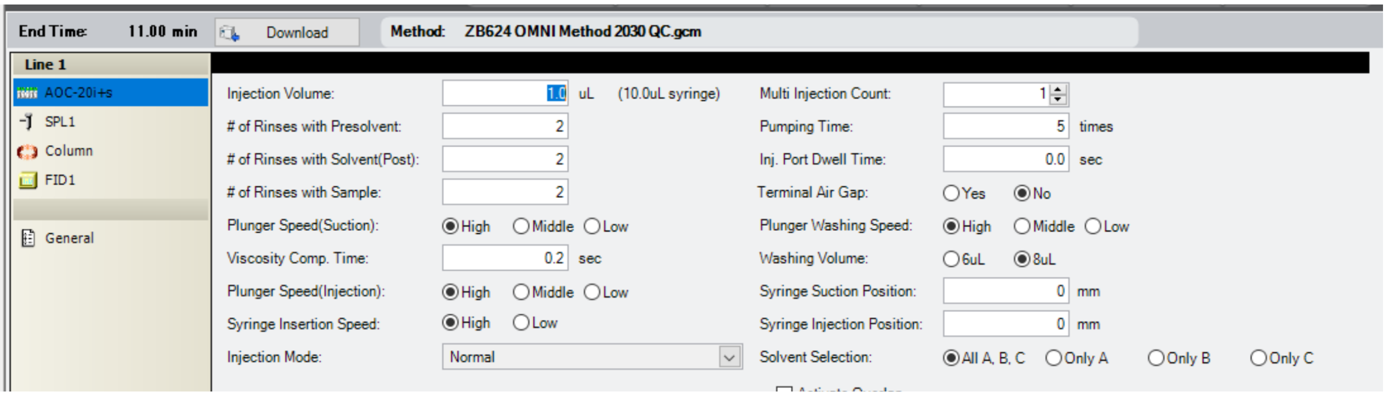

Figure S1. AOC-20i Parameters Set for OMNI in LabSolutions

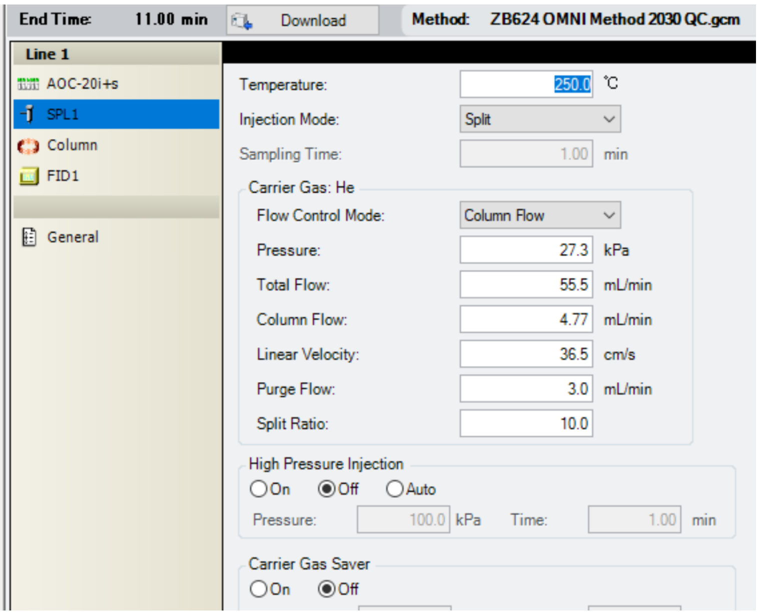

Figure S2. Split Injector Parameters Set for OMNI in LabSolution

Table S1. Ethanol Concentration Curve Data

Level	Injection	%EtOH (v/v)	Area Count	Average Area	%RSD	t_R
1	1	0.1	703277	700316	0.37	2.192
	2	0.1	698326			2.192
	3	0.1	699346			2.192
2	1	0.5	3831495	3791283	0.95	2.190
	2	0.5	3761561			2.191
	3	0.5	3780793			2.191
3	1	2.0	15622742	15628565	0.16	2.190
	2	2.0	15655204			2.190
	3	2.0	15607750			2.190
4	1	5.0	38611508	38827134	0.72	2.190
	2	5.0	39140945			2.190
	3	5.0	38728948			2.190
5	1	8.0	63050934	63682664	0.91	2.190
	2	8.0	63809867			2.190
	3	8.0	64187191			2.190
6	1	10.0	80722238	80677284	0.13	2.190
	2	10.0	80748997			2.190
	3	10.0	80560616			2.190

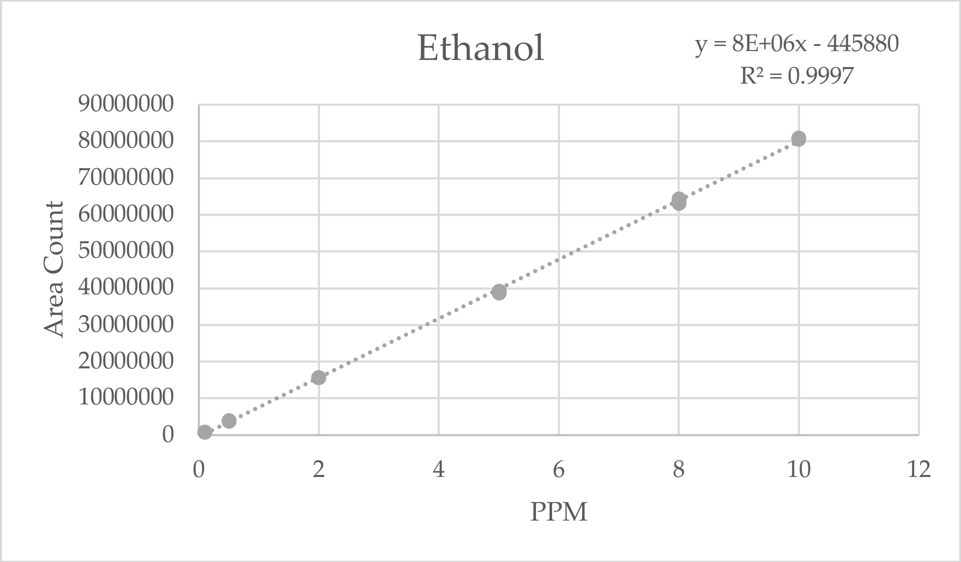

Figure S3. Ethanol Concentration Curve Graph

Table S2 . Acetone Concentration Curve

Level	Injection	Acetone (v/v)%	ppm	Area Count	Average Area	%RSD	t_R
1	1	1	50	29332	28934	1.43	2.388
	2		50	28965			2.388
	3		50	28505			2.388
2	1	5	250	145854	144089	1.08	2.385
	2		250	142907			2.385
	3		250	143506			2.385
3	1	20	1000	595395	590657	0.72	2.190
	2		1000	589459			2.190
	3		1000	587117			2.190
4	1	50	2500	1547785	1544943	0.40	2.190
	2		2500	1549220			2.190
	3		2500	1537825			2.190
5	1	80	4000	2545617	2557275	0.40	2.190
	2		4000	2564592			2.190
	3		4000	2561617			2.190
6	1	100	5000	3363774	3355232	0.39	2.190
	2		5000	3361860			2.190
	3		5000	3340062			2.190

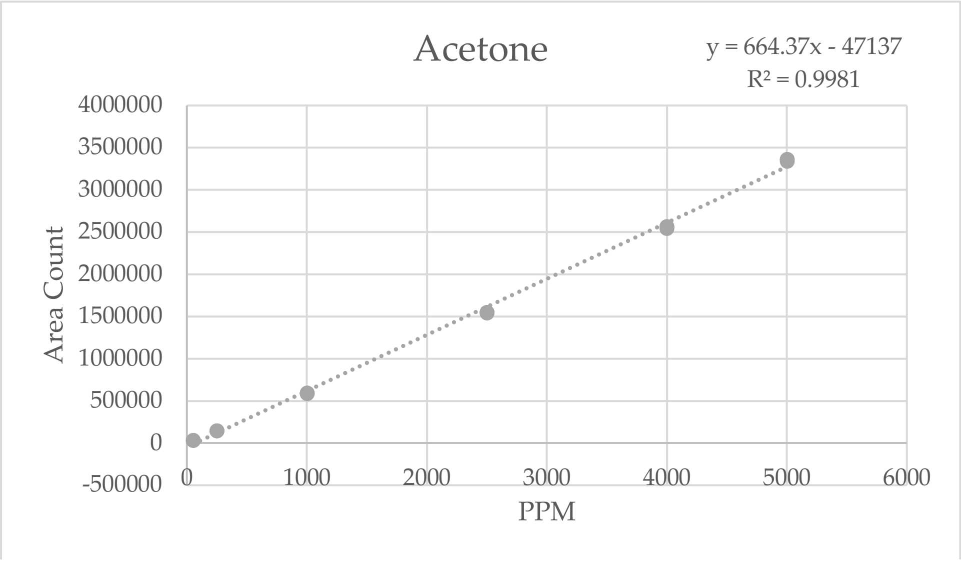

Figure S4. Acetone Concentration Curve Graph

Table S3 . Acetonitrile Concentration Curve Data

Level	Injection	MeCN (v/v)%	ppm	Area Count	Average Area	%RSD	t_R
1	1	1	4.1	4390	4322	1.48	2.527
	2		4.1	4313			2.526
	3		4.1	4263			2.527
2	1	5	20.5	17727	17154	3.86	2.524
	2		20.5	16429			2.524
	3		20.5	17306			2.524
3	1	20	82	62526	62275	0.42	2.522
	2		82	62294			2.522
	3		82	62005			2.523
4	1	50	205	156340	156158	0.55	2.522
	2		205	156914			2.522
	3		205	155221			2.522
5	1	80	328	256338	257562	0.42	2.522
	2		328	258318			2.522
	3		328	258029			2.522
6	1	100	410	339376	336544	0.77	2.522
	2		410	335953			2.522
	3		410	334304			2.522

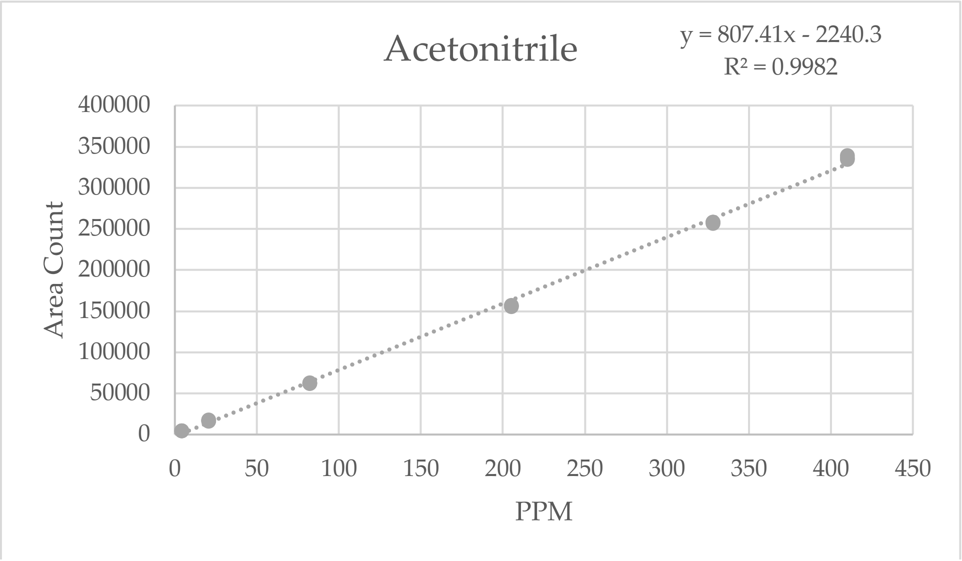

Figure S5. Acetonitrile Concentration Curve Graph

Table S4 . Triethylamine Concentration Curve

Level	Injection	Et_3N (v/v)%	ppm	Area Count	Average Area	%RSD	t_R
1	1	1	50	37610	38478	5.69	3.279
	2		50	36855			3.279
	3		50	40968			3.279
2	1	5	250	191085	184116	3.62	3.270
	2		250	183463			3.269
	3		250	177799			3.271
3	1	20	1000	770022	744576	3.31	3.268
	2		1000	742858			3.268
	3		1000	720849			3.270
4	1	50	2500	2029177	1980436	2.75	3.271
	2		2500	1990578			3.269
	3		2500	1921552			3.268
5	1	80	4000	3233842	3192442	1.46	3.269
	2		4000	3201433			3.270
	3		4000	3142052			3.270
6	1	100	5000	4486982	4432299	1.30	3.270
	2		5000	4438144			3.271
	3		5000	4371771			3.271

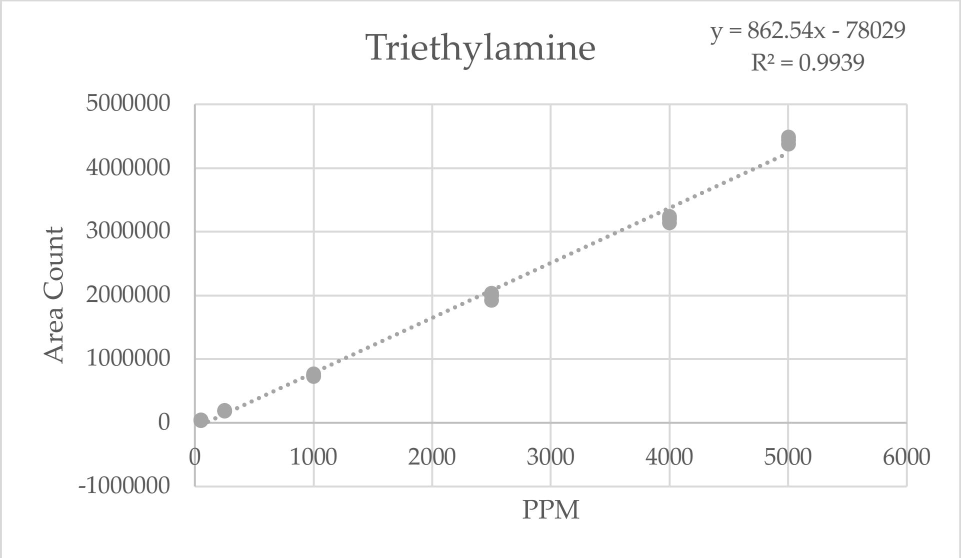

Figure S6. Triethylamine Concentration Curve Graph

Table S5 . *N,N*-dimethylformamide Concentration Curve Data

Level	Injection	DMF (v/v)%	PPM	Area Count	Average Area	%RSD	t_R
1	1	1	8.8	1272	1269	0.73	4.156
	2		8.8	1277			4.152
	3		8.8	1259			4.151
2	1	5	44	7719	7589	2.66	4.141
	2		44	7356			4.137
	3		44	7692			4.142
3	1	20	176	32268	32353	0.23	4.138
	2		176	32389			4.139
	3		176	32403			4.144
4	1	50	440	80584	81697	1.40	4.151
	2		440	82875			4.145
	3		440	81631			4.141
5	1	80	704	135523	137184	1.31	4.137
	2		704	136930			4.139
	3		704	139099			4.140
6	1	100	880	175562	173927	0.82	4.136
	2		880	172916			4.138
	3		880	173302			4.136

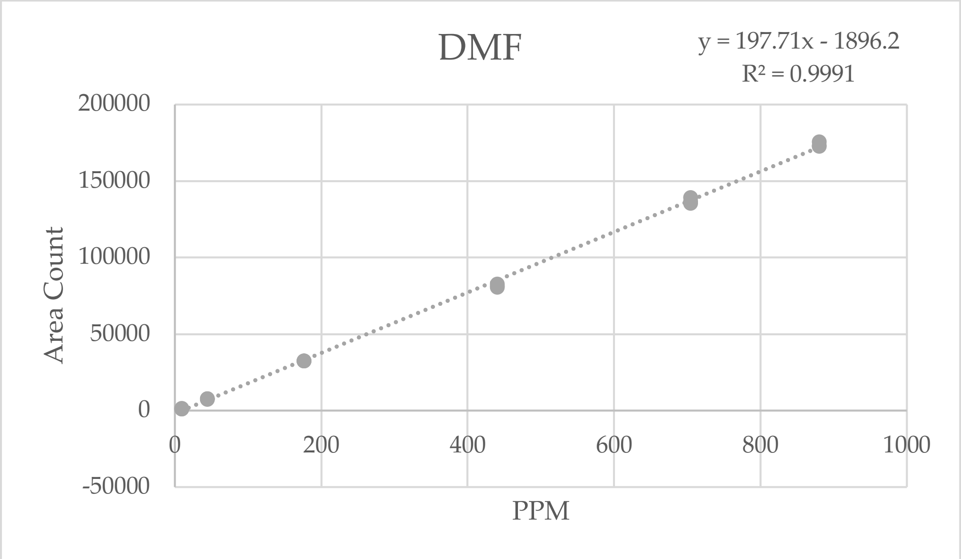

Figure S7. *N,N*-dimethylformamide Concentration Curve Graph

Table S6 . Dimethyl Sulfoxide Concentration Curve

Level	Injection	DMSO (v/v)%	PPM	Area Count	Average Area	%RSD	t_R
1	1	1	50	27278	26868	1.45	4.484
	2		50	26824			4.479
	3		50	26502			4.478
2	1	5	250	150471	149500	0.79	4.475
	2		250	148194			4.469
	3		250	149834			4.476
3	1	20	1000	646305	651449	0.69	4.474
	2		1000	653291			4.477
	3		1000	654750			4.482
4	1	50	2500	1666154	1691116	1.41	4.495
	2		2500	1713691			4.486
	3		2500	1693502			4.481
5	1	80	4000	2784153	2823538	1.32	4.477
	2		4000	2828451			4.480
	3		4000	2858010			4.481
6	1	100	5000	3533318	3527489	0.22	4.477
	2		5000	3518594			4.479
	3		5000	3530556			4.477

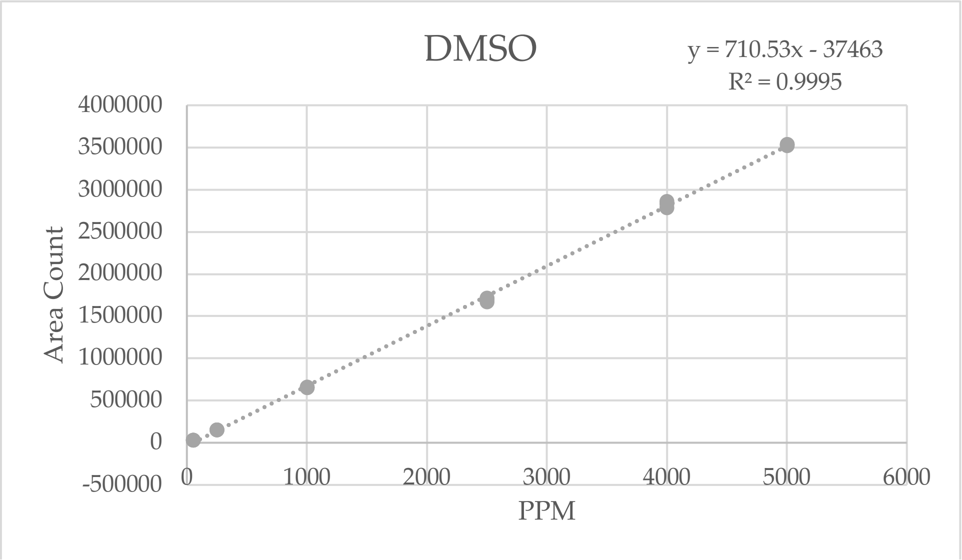

Figure S8. Dimethyl Sulfoxide Concentration Curve Graph

5/22/2023 9:53:54 AM Page 1 / 1

SHIMADZU LabSolutions Analysis Report

<Sample Information>

Sample Name : OMN-030623-01-T03
Sample ID : OMN-01-03
Data Filename : OMN-030623-01-T03.gcd
Method Filename : ZB624 OMNI Method 2030 QC.gcm
Batch Filename : OMN-CURVE-STD-TEST-01.gcb
Vial # : 7Sample Type : Standard
Injection Volume : 1 uLLevel : 1
Date Acquired : 3/6/2023 2:21:47 PMAcquired by : System Administrator
Date Processed : 3/16/2023 3:36:01 PMProcessed by : System Administrator

<Chromatogram>

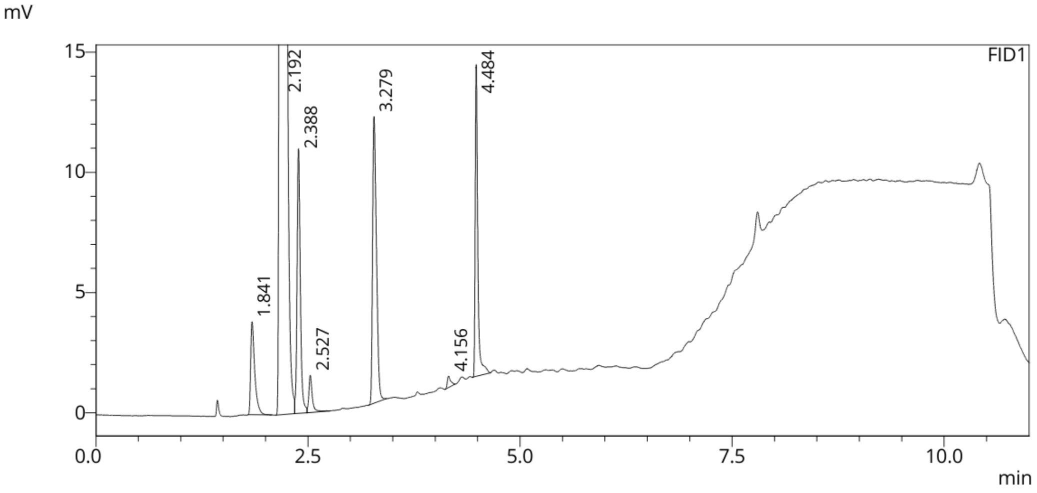

<Peak Table>

FID1

Peak#	Ret. Time	Area	Height	Conc.	Unit	Mark	Name
1	1.841	12924	3855	57.008	ppm	M	MeOH
2	2.192	703277	215613	0.143	ppm	M	EtOH
3	2.388	29332	10968	115.100	ppm	V M	Acetone
4	2.527	4390	1542	8.212	ppm	V M	ACN
5	3.279	37610	11894	134.068	ppm	M	TEA
6	4.156	1272	472	16.024	ppm	M	DMF
7	4.484	27278	12856	91.117	ppm	M	DMSO
Total		816083	257200				

C:\LabSolutions\Data\GC 2030 Data\Test Runs\OMNES\03MAR23\OMN-030623-01-T03.gcd

Figure S9. Chromatograph for Level 1 Injection 1

5/22/2023 9:54:47 AM Page 1 / 1

SHIMADZU LabSolutions

Analysis Report

<Sample Information>

Sample Name : OMN-030623-01-T04
Sample ID : OMN-01-04
Data Filename : OMN-030623-01-T04.gcd
Method Filename : ZB624 OMNI Method 2030 QC.gcm
Batch Filename : OMN-CURVE-STD-TEST-01.gcb
Vial # : 7Sample Type : Standard
Injection Volume : 1 uLLevel : 1
Date Acquired : 3/6/2023 2:36:31 PMAcquired by CA : System Administrator
Date Processed : 3/16/2023 3:36:01 PMProcessed by CA : System Administrator

<Chromatogram>

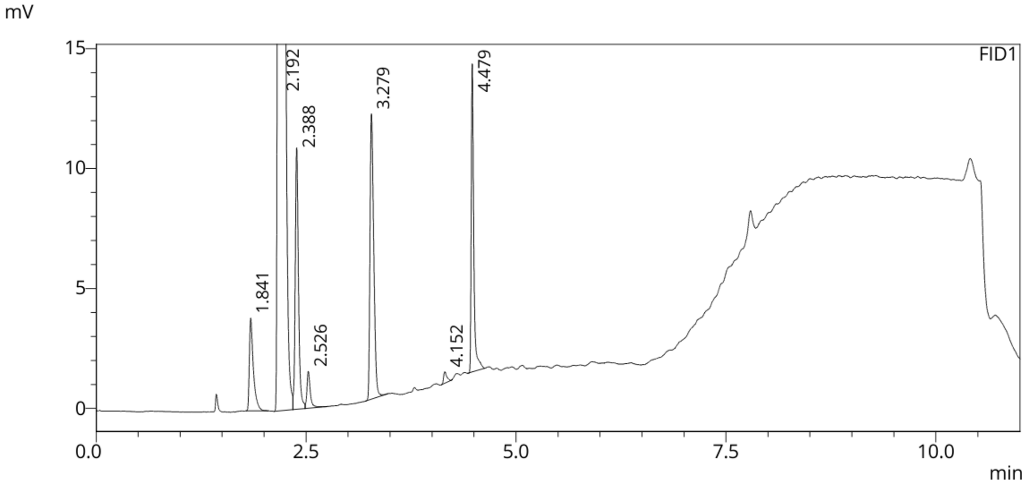

<Peak Table>

FID1

Peak#	Ret. Time	Area	Height	Conc.	Unit	Mark	Name
1	1.841	12935	3851	57.031	ppm	M	MeOH
2	2.192	698326	215388	0.142	ppm	M	EtOH
3	2.388	28965	10863	114.548	ppm	V M	Acetone
4	2.526	4313	1536	8.116	ppm	V M	ACN
5	3.279	36855	11867	133.193	ppm	M	TEA
6	4.152	1277	483	16.049	ppm	M	DMF
7	4.479	26824	12763	90.477	ppm	M	DMSO
Total		809494	256752				

C:\LabSolutions\Data\GC 2030 Data\Test Runs\OMNES\03MAR23\OMN-030623-01-T04.gcd

Figure S10. Chromatograph for Level 1 Injection 2

5/22/2023 9:55:16 AM Page 1 / 1

SHIMADZU LabSolutions

Analysis Report

<Sample Information>

Sample Name : OMN-030623-01-T05
Sample ID : OMN-01-05
Data Filename : OMN-030623-01-T05.gcd
Method Filename : ZB624 OMNI Method 2030 QC.gcm
Batch Filename : OMN-CURVE-STD-TEST-01.gcb
Vial # : 7Sample Type : Standard
Injection Volume : 1 uLLevel : 1
Date Acquired : 3/6/2023 2:51:16 PMAcquired by : System Administrator
Date Processed : 3/16/2023 3:36:01 PMProcessed by : System Administrator

<Chromatogram>

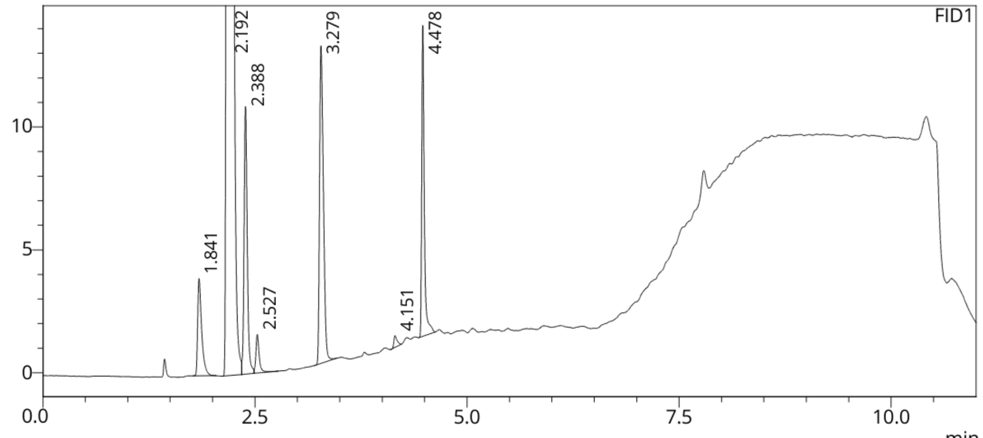

<Peak Table>

FID1

Peak#	Ret. Time	Area	Height	Conc.	Unit	Mark	Name
1	1.841	13012	3944	57.192	ppm	M	MeOH
2	2.192	699346	221348	0.142	ppm	M	EtOH
3	2.388	28505	10853	113.854	ppm	V M	Acetone
4	2.527	4263	1563	8.055	ppm	V M	ACN
5	3.279	40968	12896	137.961	ppm	M	TEA
6	4.151	1259	471	15.959	ppm	M	DMF
7	4.478	26502	12573	90.025	ppm	M	DMSO
Total		813855	263647				

C:\LabSolutions\Data\GC 2030 Data\Test Runs\OMNES\03MAR23\OMN-030623-01-T05.gcd

Figure S11. Chromatograph for Level 1 Injection 3

5/22/2023 9:57:42 AM Page 1 / 1

SHIMADZU LabSolutions

Analysis Report

<Sample Information>

Sample Name : OMN-030623-05-T02
Sample ID : OMN-05-02
Data Filename : OMN-030623-05-T02.gcd
Method Filename : ZB624 OMNI Method 2030 QC.gcm
Batch Filename : OMN-CURVE-STD-TEST-01.gcb
Vial # : 6Sample Type : Standard
Injection Volume : 1 uLLevel : 2
Date Acquired : 3/6/2023 3:06:05 PMAcquired by CA : System Administrator
Date Processed : 3/16/2023 3:36:01 PMProcessed by CA : System Administrator

<Chromatogram>

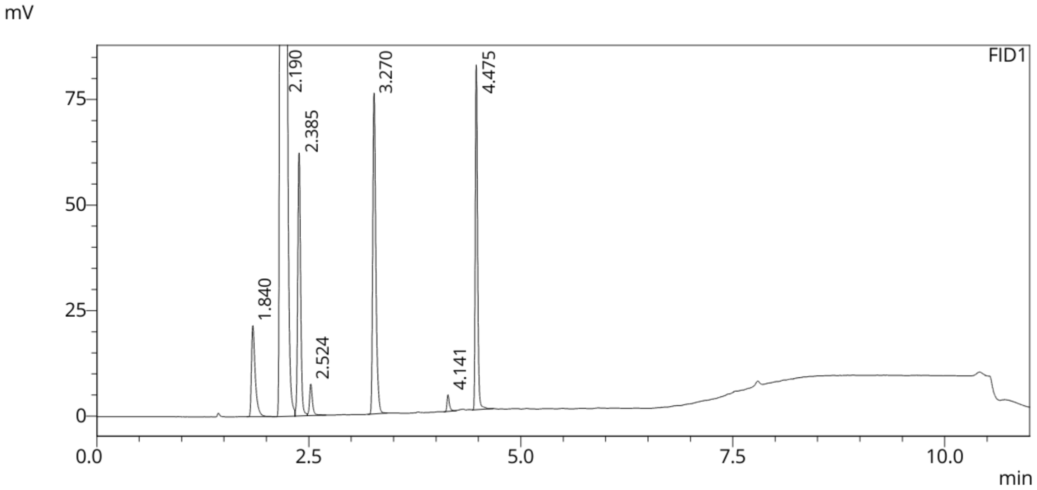

<Peak Table>

FID1

Peak#	Ret. Time	Area	Height	Conc.	Unit	Mark	Name
1	1.840	65946	21434	167.423	ppm	M	MeOH
2	2.190	3831495	1286451	0.532	ppm	M	EtOH
3	2.385	145854	61301	290.487	ppm	V M	Acetone
4	2.524	17727	7393	24.730	ppm	V M	ACN
5	3.270	191085	75707	312.002	ppm	M	TEA
6	4.141	7719	3911	48.631	ppm	M	DMF
7	4.475	150471	80359	264.500	ppm	M	DMSO
Total		4410297	1536557				

C:\LabSolutions\Data\GC 2030 Data\Test Runs\OMNES\03MAR23\OMN-030623-05-T02.gcd

Figure S12. Chromatograph for Level 2 Injection 1

5/22/2023 9:58:07 AM Page 1 / 1

SHIMADZU LabSolutions Analysis Report

<Sample Information>

Sample Name : OMN-030623-05-T03
Sample ID : OMN-05-03
Data Filename : OMN-030623-05-T03.gcd
Method Filename : ZB624 OMNI Method 2030 QC.gcm
Batch Filename : OMN-CURVE-STD-TEST-01.gcb
Vial # : 6Sample Type : Standard
Injection Volume : 1 uLLevel : 2
Date Acquired : 3/6/2023 3:20:52 PMAcquired by : System Administrator
Date Processed : 3/16/2023 3:36:01 PMProcessed by : System Administrator

<Chromatogram>

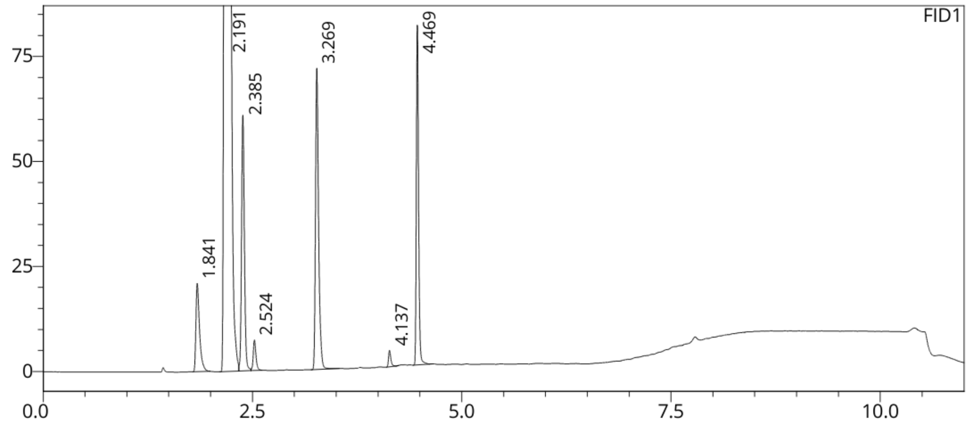

<Peak Table>

FID1

Peak#	Ret. Time	Area	Height	Conc.	Unit	Mark	Name
1	1.841	63773	20949	162.898	ppm	M	MeOH
2	2.191	3761561	1258975	0.523	ppm	M	EtOH
3	2.385	142907	59844	286.051	ppm	V M	Acetone
4	2.524	16429	7199	23.123	ppm	V M	ACN
5	3.269	183463	71433	303.165	ppm	M	TEA
6	4.137	7356	3868	46.798	ppm	M	DMF
7	4.469	148194	80207	261.296	ppm	M	DMSO
Total		4323685	1502476				

C:\LabSolutions\Data\GC 2030 Data\Test Runs\OMNES\03MAR23\OMN-030623-05-T03.gcd

Figure S13. Chromatograph for Level 2 Injection 2

5/22/2023 9:58:42 AM Page 1 / 1

SHIMADZU LabSolutions **Analysis Report**

<Sample Information>

Sample Name : OMN-030623-05-T04
Sample ID : OMN-05-04
Data Filename : OMN-030623-05-T04.gcd
Method Filename : ZB624 OMNI Method 2030 QC.gcm
Batch Filename : OMN-CURVE-STD-TEST-01.gcb
Vial # : 6Sample Type : Standard
Injection Volume : 1 uLLevel : 2
Date Acquired : 3/6/2023 3:35:38 PMAcquired by CA : System Administrator
Date Processed : 3/16/2023 3:36:01 PMProcessed by CA : System Administrator

<Chromatogram>

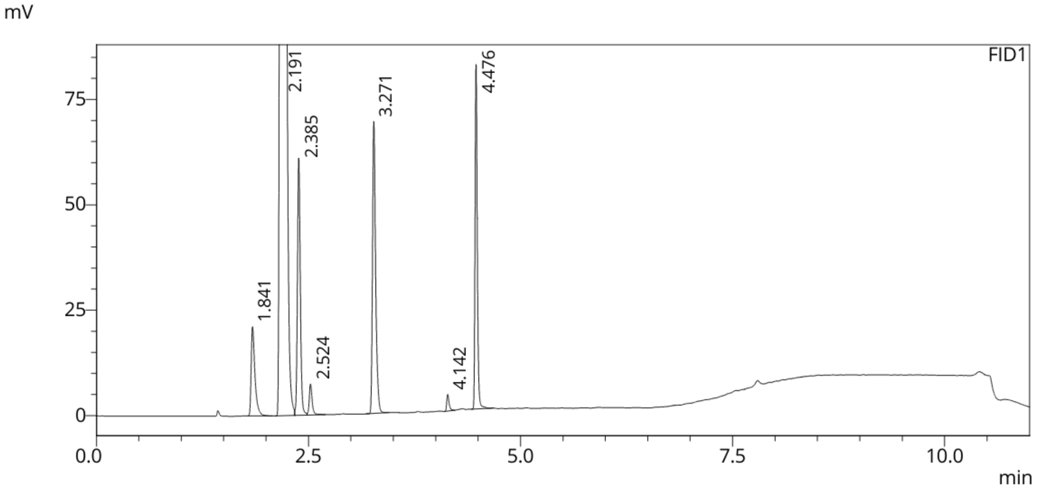

<Peak Table>

FID1

Peak#	Ret. Time	Area	Height	Conc.	Unit	Mark	Name
1	1.841	65116	21076	165.694	ppm	M	MeOH
2	2.191	3780793	1268106	0.525	ppm	M	EtOH
3	2.385	143506	59850	286.953	ppm	V M	Acetone
4	2.524	17306	7287	24.208	ppm	V M	ACN
5	3.271	177799	68886	296.599	ppm	M	TEA
6	4.142	7692	3899	48.496	ppm	M	DMF
7	4.476	149834	79570	263.603	ppm	M	DMSO
Total		4342046	1508673				

C:\LabSolutions\Data\GC 2030 Data\Test Runs\OMNES\03MAR23\OMN-030623-05-T04.gcd

Figure S14. Chromatograph for Level 2 Injection 3

5/22/2023 9:59:15 AM Page 1 / 1

SHIMADZU LabSolutions

Analysis Report

<Sample Information>

Sample Name : OMN-030623-20-T02
Sample ID : OMN-20-02
Data Filename : OMN-030623-20-T02.gcd
Method Filename : ZB624 OMNI Method 2030 QC.gcm
Batch Filename : OMN-CURVE-STD-TEST-01.gcb
Vial # : 5Sample Type : Standard
Injection Volume : 1 uLLevel : 3
Date Acquired : 3/6/2023 4:05:09 PMAcquired by : System Administrator
Date Processed : 3/16/2023 3:36:01 PMProcessed by : System Administrator

<Chromatogram>

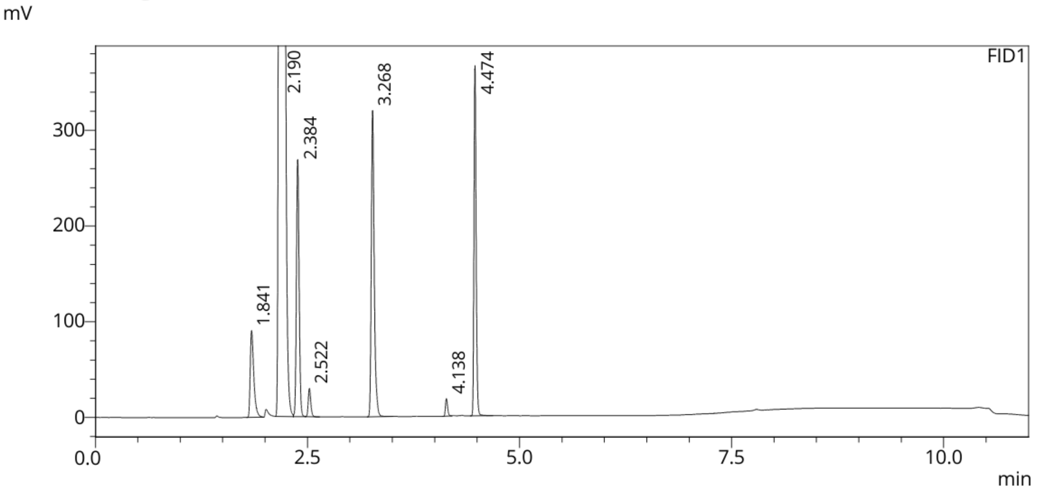

<Peak Table>

FID1

Peak#	Ret. Time	Area	Height	Conc.	Unit	Mark	Name
1	1.841	264117	90530	580.098	ppm	M	MeOH
2	2.190	15622742	5390899	1.997	ppm	M	EtOH
3	2.384	595395	265772	967.128	ppm	V M	Acetone
4	2.522	62526	29588	80.215	ppm	V M	ACN
5	3.268	770022	316839	983.201	ppm	M	TEA
6	4.138	32268	18128	172.803	ppm	M	DMF
7	4.474	646305	362264	962.341	ppm	M	DMSO
Total		17993376	6474019				

C:\LabSolutions\Data\GC 2030 Data\Test Runs\OMNES\03MAR23\OMN-030623-20-T02.gcd

Figure S15. Chromatograph for Level 3 Injection 1

5/22/2023 9:59:36 AM Page 1 / 1

SHIMADZU LabSolutions **Analysis Report**

<Sample Information>

Sample Name : OMN-030623-20-T03
Sample ID : OMN-20-03
Data Filename : OMN-030623-20-T03.gcd
Method Filename : ZB624 OMNI Method 2030 QC.gcm
Batch Filename : OMN-CURVE-STD-TEST-01.gcb
Vial # : 5Sample Type : Standard
Injection Volume : 1 uLLevel : 3
Date Acquired : 3/6/2023 4:19:55 PMAcquired by : System Administrator
Date Processed : 3/16/2023 3:36:01 PMProcessed by : System Administrator

<Chromatogram>

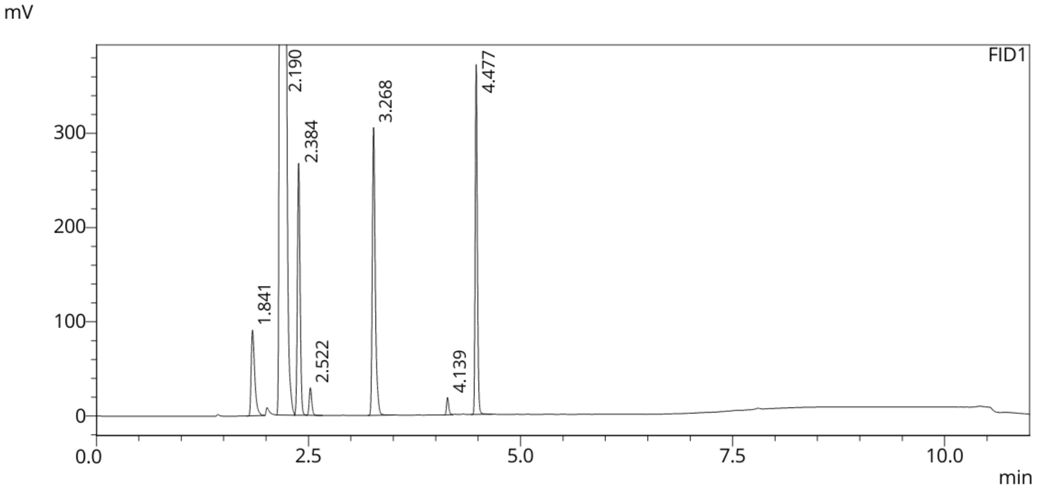

<Peak Table>

FID1

Peak#	Ret. Time	Area	Height	Conc.	Unit	Mark	Name
1	1.841	263127	90456	578.035	ppm	M	MeOH
2	2.190	15655204	5384320	2.001	ppm	M	EtOH
3	2.384	589459	264976	958.193	ppm	V M	Acetone
4	2.522	62294	29274	79.928	ppm	V M	ACN
5	3.268	742858	302743	951.708	ppm	M	TEA
6	4.139	32389	17855	173.414	ppm	M	DMF
7	4.477	653291	365622	972.173	ppm	M	DMSO
Total		17998622	6455246				

C:\LabSolutions\Data\GC 2030 Data\Test Runs\OMNES\03MAR23\OMN-030623-20-T03.gcd

Figure S16. Chromatograph for Level 3 Injection 2

5/22/2023 9:59:58 AM Page 1 / 1

SHIMADZU LabSolutions **Analysis Report**

<Sample Information>

Sample Name : OMN-030623-20-T04
Sample ID : OMN-20-04
Data Filename : OMN-030623-20-T04.gcd
Method Filename : ZB624 OMNI Method 2030 QC.gcm
Batch Filename : OMN-CURVE-STD-TEST-01.gcb
Vial # : 5Sample Type : Standard
Injection Volume : 1 uLLevel : 3
Date Acquired : 3/6/2023 4:34:43 PMAcquired by CA : System Administrator
Date Processed : 3/16/2023 3:36:01 PMProcessed by CA : System Administrator

<Chromatogram>

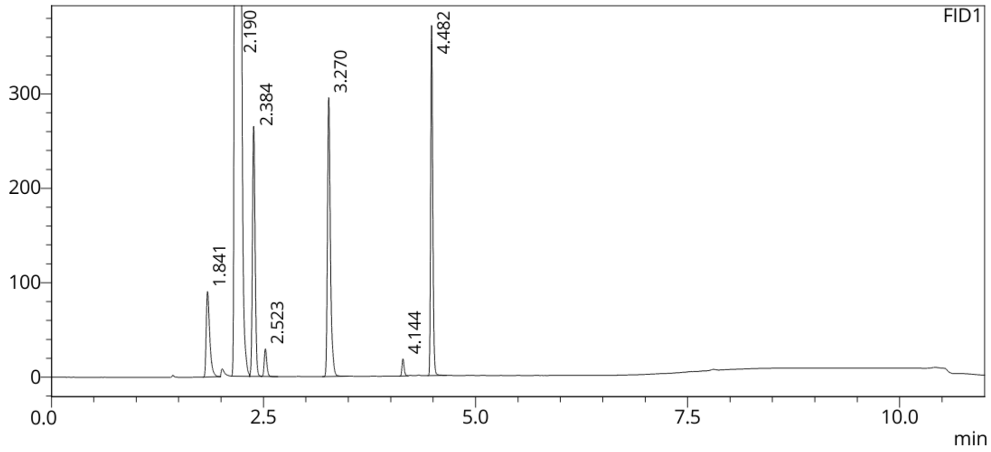

<Peak Table>

FID1

Peak#	Ret. Time	Area	Height	Conc.	Unit	Mark	Name
1	1.841	262302	90330	576.318	ppm	M	MeOH
2	2.190	15607750	5366837	1.995	ppm	M	EtOH
3	2.384	587117	261863	954.668	ppm	V M	Acetone
4	2.523	62005	29190	79.569	ppm	V M	ACN
5	3.270	720849	294092	926.192	ppm	M	TEA
6	4.144	32403	17804	173.484	ppm	M	DMF
7	4.482	654750	368062	974.226	ppm	M	DMSO
Total		17927176	6428178				

C:\LabSolutions\Data\GC 2030 Data\Test Runs\OMNES\03MAR23\OMN-030623-20-T04.gcd

Figure S17. Chromatograph for Level 3 Injection 3

5/22/2023 10:02:02 AM Page 1 / 1

SHIMADZU LabSolutions **Analysis Report**

<Sample Information>

Sample Name : OMN-030623-50-T03
Sample ID : OMN-50-03
Data Filename : OMN-030623-50-T03.gcd
Method Filename : ZB624 OMNI Method 2030 QC.gcm
Batch Filename : OMN-CURVE-STD-TEST-01.gcb
Vial # : 3Sample Type : Standard
Injection Volume : 1 uLLevel : 4
Date Acquired : 3/6/2023 5:19:12 PMAcquired by CA : System Administrator
Date Processed : 3/16/2023 3:36:02 PMProcessed by CA : System Administrator

<Chromatogram>

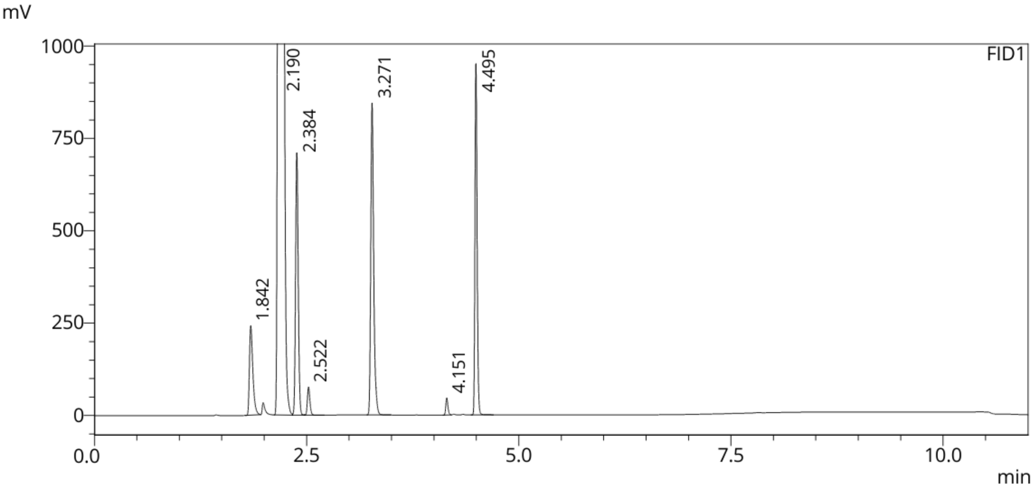

<Peak Table>

FID1

Peak#	Ret. Time	Area	Height	Conc.	Unit	Mark	Name
1	1.842	676487	241553	1438.824	ppm	M	MeOH
2	2.190	38611508	13597928	4.854	ppm	M	EtOH
3	2.384	1547785	702220	2400.648	ppm	V M	Acetone
4	2.522	156340	75969	196.407	ppm	V M	ACN
5	3.271	2029177	836092	2443.022	ppm	M	TEA
6	4.151	80584	45732	417.182	ppm	M	DMF
7	4.495	1666154	934275	2397.683	ppm	M	DMSO
Total		44768036	16433769				

C:\LabSolutions\Data\GC 2030 Data\Test Runs\OMNES\03MAR23\OMN-030623-50-T03.gcd

Figure S18. Chromatograph for Level 4 Injection 1

5/22/2023 10:02:35 AM Page 1 / 1

SHIMADZU LabSolutions **Analysis Report**

<Sample Information>

Sample Name : OMN-030623-50-T04
Sample ID : OMN-50-04
Data Filename : OMN-030623-50-T04.gcd
Method Filename : ZB624 OMNI Method 2030 QC.gcm
Batch Filename : OMN-CURVE-STD-TEST-01.gcb
Vial # : 3Sample Type : Standard
Injection Volume : 1 uLLevel : 4
Date Acquired : 3/6/2023 5:34:02 PMAcquired by : System Administrator
Date Processed : 3/16/2023 3:36:01 PMProcessed by : System Administrator

<Chromatogram>

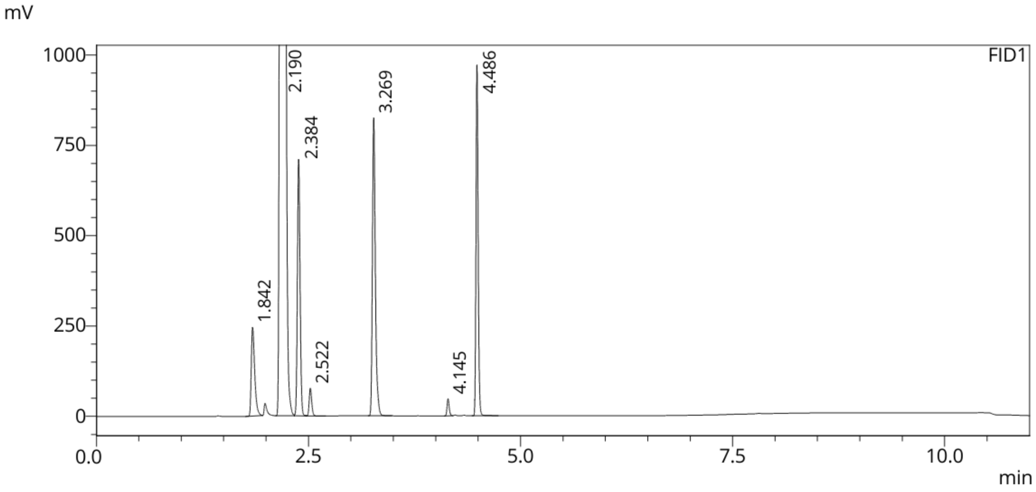

<Peak Table>

FID1

Peak#	Ret. Time	Area	Height	Conc.	Unit	Mark	Name
1	1.842	685536	245475	1457.668	ppm	M	MeOH
2	2.190	39140945	13839848	4.920	ppm	M	EtOH
3	2.384	1549220	703839	2402.808	ppm	V M	Acetone
4	2.522	156914	76263	197.117	ppm	V M	ACN
5	3.269	1990578	821911	2398.272	ppm	M	TEA
6	4.145	82875	46413	428.772	ppm	M	DMF
7	4.486	1713691	958286	2464.587	ppm	M	DMSO
Total		45319760	16692037				

C:\LabSolutions\Data\GC 2030 Data\Test Runs\OMNES\03MAR23\OMN-030623-50-T04.gcd

Figure S19. Chromatograph for Level 4 Injection 2

5/22/2023 10:03:03 AM Page 1 / 1

SHIMADZU LabSolutions Analysis Report

<Sample Information>

Sample Name : OMN-030623-50-T05
Sample ID : OMN-50-05
Data Filename : OMN-030623-50-T05.gcd
Method Filename : ZB624 OMNI Method 2030 QC.gcm
Batch Filename : OMN-CURVE-STD-TEST-01.gcb
Vial # : 3Sample Type : Standard
Injection Volume : 1 uLLevel : 4
Date Acquired : 3/6/2023 5:48:54 PMAcquired by : System Administrator
Date Processed : 3/16/2023 3:36:01 PMProcessed by CA : System Administrator

<Chromatogram>

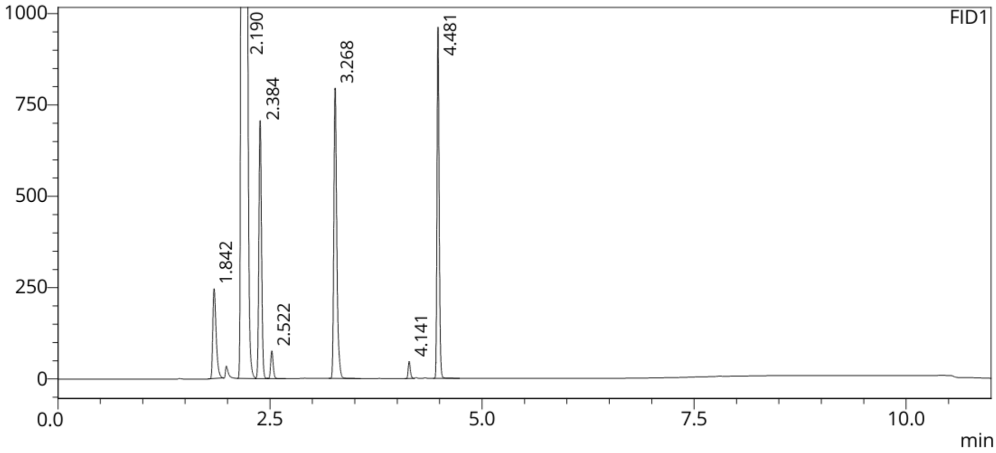

<Peak Table>

FID1

Peak#	Ret. Time	Area	Height	Conc.	Unit	Mark	Name
1	1.842	675753	245278	1437.296	ppm	M	MeOH
2	2.190	38728948	13784339	4.869	ppm	M	EtOH
3	2.384	1537825	699894	2385.657	ppm	V M	Acetone
4	2.522	155221	75477	195.021	ppm	V M	ACN
5	3.268	1921552	790383	2318.246	ppm	M	TEA
6	4.141	81631	46180	422.476	ppm	M	DMF
7	4.481	1693502	930625	2436.173	ppm	M	DMSO
Total		44794433	16572176				

C:\LabSolutions\Data\GC 2030 Data\Test Runs\OMNES\03MAR23\OMN-030623-50-T05.gcd

Figure S20. Chromatograph for Level 4 Injection 3

5/22/2023 10:03:38 AM Page 1 / 1

SHIMADZU LabSolutions

Analysis Report

<Sample Information>

Sample Name : OMN-030623-80-T02
Sample ID : OMN-80-02
Data Filename : OMN-030623-80-T02.gcd
Method Filename : ZB624 OMNI Method 2030 QC.gcm
Batch Filename : OMN-CURVE-STD-TEST-01.gcb
Vial # : 4Sample Type : Standard
Injection Volume : 1 uLLevel : 5
Date Acquired : 3/6/2023 6:03:44 PMAcquired by CA : System Administrator
Date Processed : 3/16/2023 3:36:02 PMProcessed by CA : System Administrator

<Chromatogram>

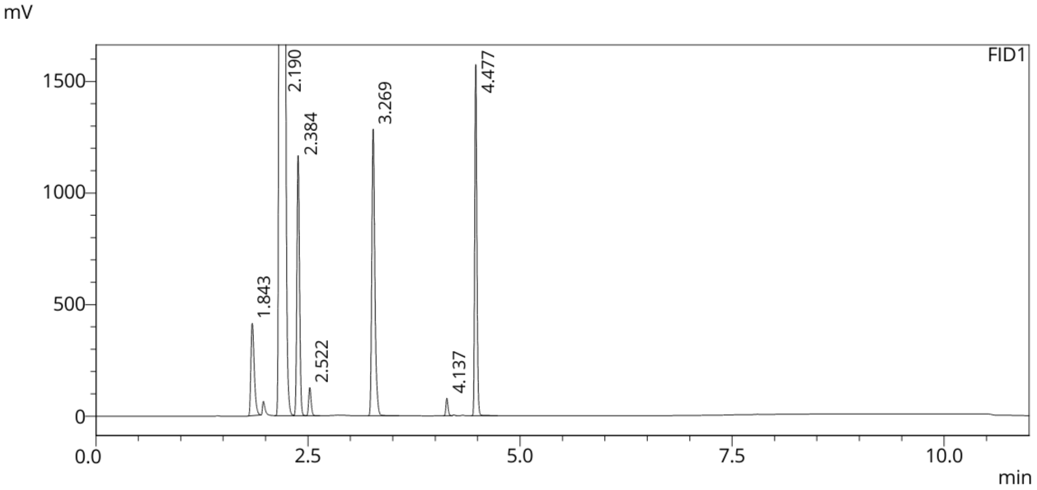

<Peak Table>

FID1

Peak#	Ret. Time	Area	Height	Conc.	Unit	Mark	Name
1	1.843	1136693	411133	2397.163	ppm	M	MeOH
2	2.190	63050934	22544936	7.892	ppm	M	EtOH
3	2.384	2545617	1155797	3902.567	ppm	V M	Acetone
4	2.522	256338	125632	320.257	ppm	V M	ACN
5	3.269	3233842	1279926	3839.669	ppm	M	TEA
6	4.137	135523	78076	695.061	ppm	M	DMF
7	4.477	2784153	1560035	3971.162	ppm	M	DMSO
Total		73143099	27155535				

C:\LabSolutions\Data\GC 2030 Data\Test Runs\OMNES\03MAR23\OMN-030623-80-T02.gcd

Figure 21. Chromatograph for Level 5 Injection 1

5/22/2023 10:04:01 AM Page 1 / 1

SHIMADZU LabSolutions

Analysis Report

<Sample Information>

Sample Name : OMN-030623-80-T03
Sample ID : OMN-80-03
Data Filename : OMN-030623-80-T03.gcd
Method Filename : ZB624 OMNI Method 2030 QC.gcm
Batch Filename : OMN-CURVE-STD-TEST-01.gcb
Vial # : 4
Sample Type : Standard
Injection Volume : 1 uL
Level : 5
Date Acquired : 3/6/2023 6:18:32 PM
Acquired by : System Administrator
Date Processed : 3/16/2023 3:36:02 PM
Processed by : System Administrator

<Chromatogram>

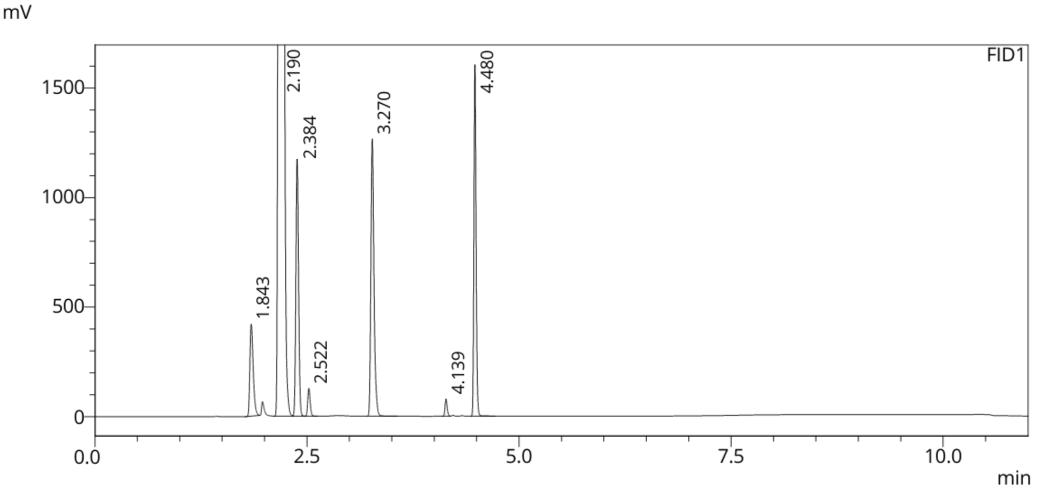

<Peak Table>

FID1

Peak#	Ret. Time	Area	Height	Conc.	Unit	Mark	Name
1	1.843	1143912	417091	2412.197	ppm	M	MeOH
2	2.190	63809867	22865174	7.986	ppm	M	EtOH
3	2.384	2564592	1164503	3931.129	ppm	V M	Acetone
4	2.522	258318	126629	322.709	ppm	V M	ACN
5	3.270	3201433	1261643	3802.095	ppm	M	TEA
6	4.139	136930	78372	702.181	ppm	M	DMF
7	4.480	2828451	1577645	4033.509	ppm	M	DMSO
Total		73943503	27491056				

C:\LabSolutions\Data\GC 2030 Data\Test Runs\OMNES\03MAR23\OMN-030623-80-T03.gcd

Figure S22. Chromatograph for Level 5 Injection 2

5/22/2023 10:04:27 AM Page 1 / 1

SHIMADZU LabSolutions

Analysis Report

<Sample Information>

Sample Name : OMN-030623-80-T04
Sample ID : OMN-80-04
Data Filename : OMN-030623-80-T04.gcd
Method Filename : ZB624 OMNI Method 2030 QC.gcm
Batch Filename : OMN-CURVE-STD-TEST-01.gcb
Vial # : 4Sample Type : Standard
Injection Volume : 1 uLLevel : 5
Date Acquired : 3/6/2023 6:33:18 PMAcquired by : System Administrator
Date Processed : 3/16/2023 3:36:02 PMProcessed by : System Administrator

<Chromatogram>

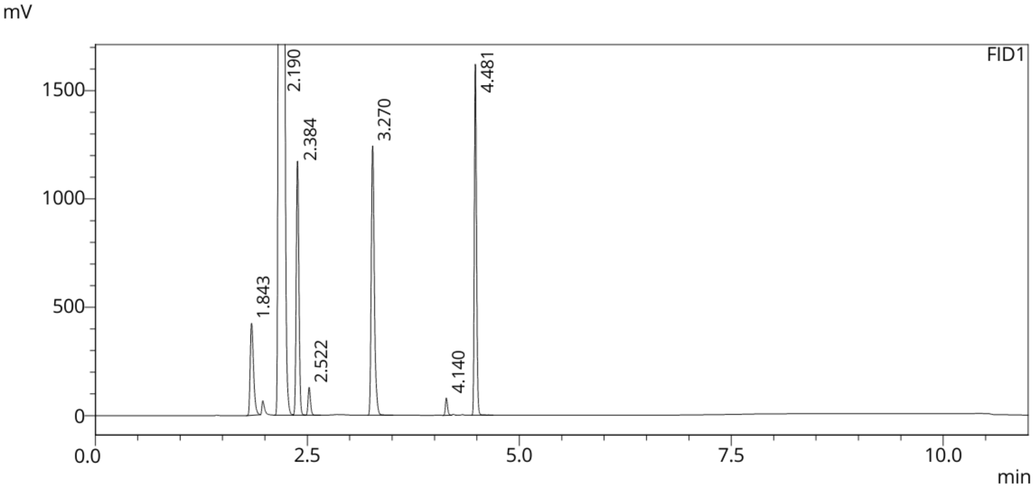

<Peak Table>

FID1

Peak#	Ret. Time	Area	Height	Conc.	Unit	Mark	Name
1	1.843	1160962	422030	2447.701	ppm	M	MeOH
2	2.190	64187191	23121428	8.033	ppm	M	EtOH
3	2.384	2561617	1162947	3926.650	ppm	V M	Acetone
4	2.522	258029	127138	322.351	ppm	V M	ACN
5	3.270	3142052	1237210	3733.251	ppm	M	TEA
6	4.140	139099	77736	713.150	ppm	M	DMF
7	4.481	2858010	1597579	4075.110	ppm	M	DMSO
Total		74306959	27746068				

C:\LabSolutions\Data\GC 2030 Data\Test Runs\OMNES\03MAR23\OMN-030623-80-T04.gcd

Figure S23. Chromatograph for Level 5 Injection 3

5/22/2023 10:04:53 AM Page 1 / 1

SHIMADZU LabSolutions

Analysis Report

<Sample Information>

Sample Name : OMN-030623-100-T01
Sample ID : OMN-100-01
Data Filename : OMN-030623-100-T01.gcd
Method Filename : ZB624 OMNI Method 2030 QC.gcm
Batch Filename : OMN-CURVE-STD-TEST-01.gcb
Vial # : 2Sample Type : Standard
Injection Volume : 1 uLLevel : 6
Date Acquired : 3/6/2023 7:02:54 PMAcquired by CA : System Administrator
Date Processed : 3/16/2023 3:36:02 PMProcessed by CA : System Administrator

<Chromatogram>

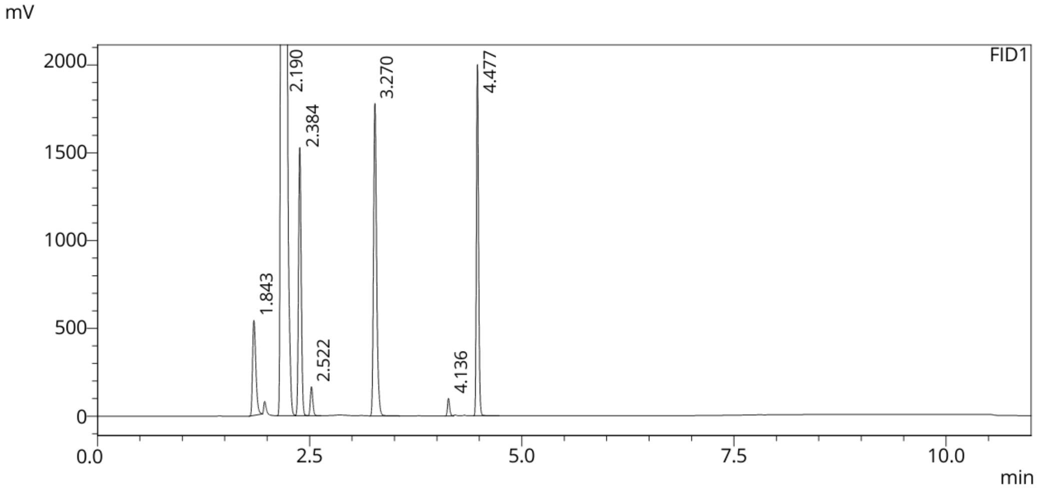

<Peak Table>

FID1

Peak#	Ret. Time	Area	Height	Conc.	Unit	Mark	Name
1	1.843	1448227	534819	3045.909	ppm	M	MeOH
2	2.190	80722238	28654006	10.088	ppm		EtOH
3	2.384	3363774	1514114	5134.044	ppm	V	Acetone
4	2.522	339376	164757	423.102	ppm	V	ACN
5	3.270	4486982	1774048	5292.516	ppm		TEA
6	4.136	175562	99390	897.576	ppm	M	DMF
7	4.477	3533318	1979573	5025.544	ppm		DMSO
Total		94069479	34720707				

C:\LabSolutions\Data\GC 2030 Data\Test Runs\OMNES\03MAR23\OMN-030623-100-T01.gcd

Figure S24. Chromatograph for Level 6 Injection 1

5/22/2023 10:05:18 AM Page 1 / 1

Analysis Report

<Sample Information>

Sample Name : OMN-030623-100-T02
Sample ID : OMN-100-02
Data Filename : OMN-030623-100-T02.gcd
Method Filename : ZB624 OMNI Method 2030 QC.gcm
Batch Filename : OMN-CURVE-STD-TEST-01.gcb
Vial # : 2Sample Type : Standard
Injection Volume : 1 uLLevel : 6
Date Acquired : 3/6/2023 7:17:40 PMAcquired by : System Administrator
Date Processed : 3/16/2023 3:36:02 PMProcessed by : System Administrator

<Chromatogram>

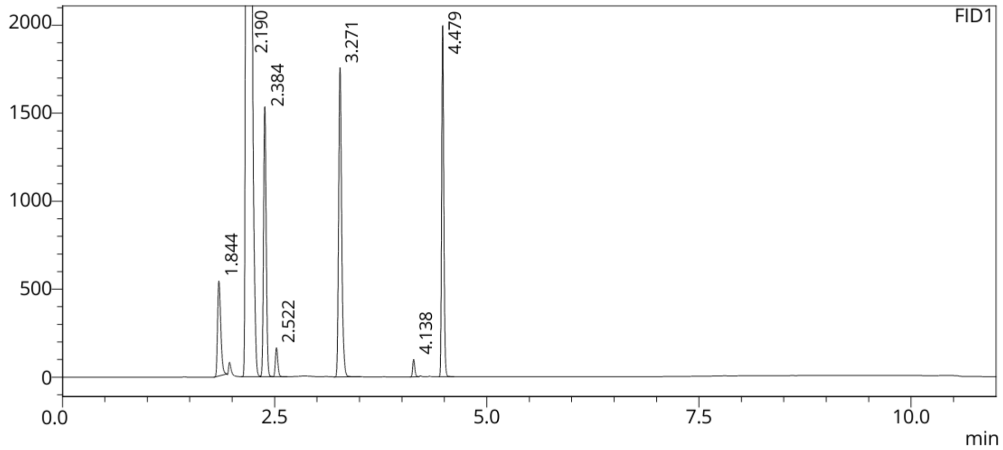

<Peak Table>

FID1

Peak#	Ret. Time	Area	Height	Conc.	Unit	Mark	Name
1	1.844	1422393	533857	2992.110	ppm	M	MeOH
2	2.190	80748997	28852551	10.091	ppm	M	EtOH
3	2.384	3361860	1518044	5131.162	ppm	V M	Acetone
4	2.522	335953	164893	418.862	ppm	V M	ACN
5	3.271	4438144	1746277	5235.896	ppm	M	TEA
6	4.138	172916	99481	884.194	ppm	M	DMF
7	4.479	3518594	1966414	5004.821	ppm	M	DMSO
Total		93998857	34881516				

C:\LabSolutions\Data\GC 2030 Data\Test Runs\OMNES\03MAR23\OMN-030623-100-T02.gcd

Figure S25. Chromatograph for Level 6 Injection 2

5/22/2023 10:05:40 AM Page 1 / 1

SHIMADZU LabSolutions **Analysis Report**

<Sample Information>

Sample Name : OMN-030623-100-T03
Sample ID : OMN-100-03
Data Filename : OMN-030623-100-T03.gcd
Method Filename : ZB624 OMNI Method 2030 QC.gcm
Batch Filename : OMN-CURVE-STD-TEST-01.gcb
Vial # : 2Sample Type : Standard
Injection Volume : 1 uLLevel : 6
Date Acquired : 3/6/2023 7:32:24 PMAcquired by CA : System Administrator
Date Processed : 3/16/2023 3:36:02 PMProcessed by CA : System Administrator

<Chromatogram>

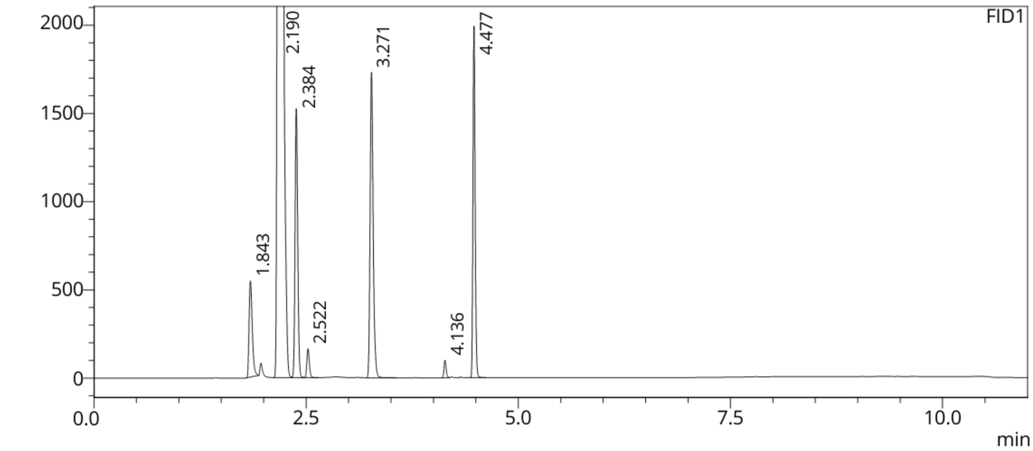

<Peak Table>

FID1

Peak#	Ret. Time	Area	Height	Conc.	Unit	Mark	Name
1	1.843	1430712	539502	3009.435	ppm	M	MeOH
2	2.190	80560616	28932853	10.068	ppm	M	EtOH
3	2.384	3340062	1509677	5098.353	ppm	V M	Acetone
4	2.522	334304	164075	416.820	ppm	V M	ACN
5	3.271	4371771	1720796	5158.945	ppm		TEA
6	4.136	173302	99159	886.148	ppm	M	DMF
7	4.477	3530556	1974237	5021.655	ppm	M	DMSO
Total		93741323	34940298				

C:\LabSolutions\Data\GC 2030 Data\Test Runs\OMNES\03MAR23\OMN-030623-100-T03.gcd

Figure S26. Chromatograph for Level 6 Injection 3

9/25/2023 9:07:36 AM Page 1 / 1

SHIMADZU LabSolutions **Analysis Report**

<Sample Information>

Sample Name : BLK-OMN-030623-01
Sample ID : BLK-01
Data Filename : BLK-OMN-030623-01.gcd
Method Filename : ZB624 OMNI Method 2030 QC.gcm
Batch Filename : OMN-CURVE-STD-TEST-01.gcb
Vial # : 1Sample Type : Unknown
Injection Volume : 1 uL
Date Acquired : 3/6/2023 12:12:28 PMAcquired by : System Administrator
Date Processed : 3/6/2023 12:23:28 PMProcessed by : System Administrator

<Chromatogram>

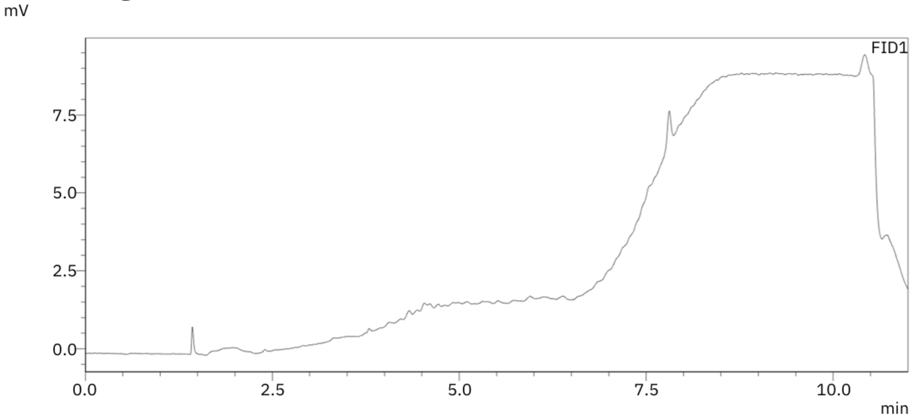

<Peak Table>

FID1

Peak#	Ret. Time	Area	Height	Conc.	Unit	Mark	Name
Total							

C:\LabSolutions\Data\GC 2030 Data\Test Runs\OMNES\03MAR23\BLK-OMN-030623-01.gcd

Figure S27. Chromatograph for Blank-01 Water Injection After OMNI

9/25/2023 9:08:07 AM Page 1 / 1

SHIMADZU LabSolutions **Analysis Report**

<Sample Information>

Sample Name	: BLK-OMN-030623-02		
Sample ID	: BLK-02		
Data Filename	: BLK-OMN-030623-02.gcd		
Method Filename	: ZB624 OMNI Method 2030 QC.gcm		
Batch Filename	: OMN-CURVE-STD-TEST-01.gcb		
Vial #	: 1	Sample Type	: Unknown
Injection Volume	: 1 uL		
Date Acquired	: 3/6/2023 1:46:01 PM	Acquired by	: System Administrator
Date Processed	: 3/6/2023 1:57:03 PM	Processed by	: System Administrator

<Chromatogram>

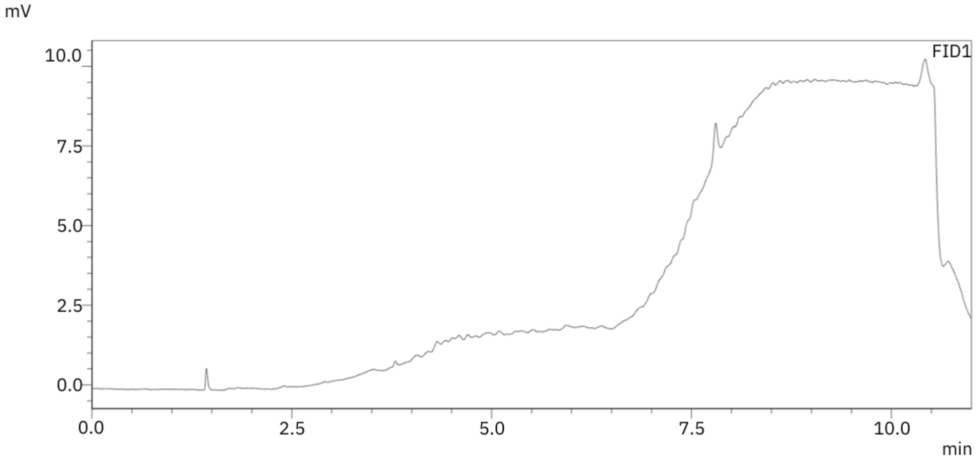

<Peak Table>

FID1

Peak#	Ret. Time	Area	Height	Conc.	Unit	Mark	Name
Total							

C:\LabSolutions\Data\GC 2030 Data\Test Runs\OMNES\03MAR23\BLK-OMN-030623-02.gcd

Figure S28. Chromatograph for Blank-02 Water Injection After OMNI

Table S7. System suitability performed on GC-2014 with original gas chromatography method for [11C]ER-176 using standard containing only acetone, ethanol, acetonitrile and DMSO.

GC System ID: CAI2R.EQ.008 (GC-2014)				
**Injection**	**AUC Acetone**	**AUC Ethanol**	**AUC Acetonitrile**	**AUC DMSO**
Injection 1	2363089	56302160	246167	2186553
Injection 2	2378290	56640406	248996	2207505
Injection 3	2365217	56594393	246111	2220736
**Average**	**2368865**	**56594393**	**247091**	**2204931**
**% RSD (≤ 5.0%)**	**0.35**	**0.32**	**0.67**	**0.78**
Confirm system suitability meets requirements.				yes

Table S8. System suitability performed on GC-2030 with new met method using standard containing only acetone, ethanol, acetonitrile and DMSO.

GC System ID: CAI2R.EQ.053 (GC-2030)				
**Injection**	**AUC Acetone**	**AUC Ethanol**	**AUC Acetonitrile**	**AUC DMSO**
Injection 1	3676891	85367255	383785	3720849
Injection 2	3711201	85955739	387925	3696464
Injection 3	3685153	85528565	385482	3702429
**Average**	**3691082**	**85617186**	**385731**	**3706581**
**% RSD (≤ 5.0%)**	**0.49**	**0.36**	**0.54**	**0.34**
Confirm system suitability meets requirements.				yes

Table S9. Results from injectable [11C]ER-176 Sample using original ZB624 ER-176 method QC Method

**Method: ZB624 ER-176 QC Method**				
**Sample: 11C-ER176-101822-01**				
**Analyte**	**Acetone**	**EtOH**	**MeCN**	**DMSO**
System Suit. Avg. Counts	2368865	56594393	247091	2204931
Sample Counts	0.00	33676323	13751	0.00
Sample: S.S. Ratio %	0.00	0.59	0.05	0.00

Table S10. Results from injectable [11C]ER-176 Sample using OMNI Method 2030 QC

**Method: ZB624 OMNI Method 2030 QC**				
**Sample: 11C-ER176-101822-01**				
**Analyte**	**Acetone**	**EtOH**	**MeCN**	**DMSO**
System Suit. Avg. Counts	3691082	85417186	385731	3706581
Sample Counts	0.0	48949187	19459	0.0
Sample: S.S. Ratio %	0.00	0.57	0.05	0.00

Table S11. Method comparison and cross-validation results for [11C]ER-176

.

Analyte	Acetone	EtOH	MeCN	DMSO
Sample Ratio %<br>ZB624 ER-176 QC Method	0.00	0.59	0.05	0.00
Sample Ratio %<br>ZB624 OMNI QC Method	0.00	0.57	0.05	0.00
Difference between ratio %	0.00	0.02	0.00	0.00
Difference ≤ 5%?	yes	yes	yes	yes

Table S12. System suitability performed on GC-2014 with original gas chromatography method for [11C]PiB and [18F]Fallypride using standard containing only acetone, ethanol, acetonitrile and triethyl amine.

**GC System ID: CAI2R.EQ.008 (GC-2014)**				
**Injection**	**AUC**<br>**Acetone**	**AUC**<br>**Ethanol**	**AUC**<br>**Acetonitrile**	**AUC**<br>**Triethyl amine**
Injection 1	2278980	54036548	235863	3306972
Injection 2	2252740	53533881	233691	3262785
Injection 3	2242291	53245483	231733	3240242
**Average**	**2258004**	**53605304**	**233762**	**3270000**
**% RSD (≤ 5.0%)**	**0.8**	**0.7**	**0.9**	**1.0**
Confirm system suitability meets requirements.				yes

Table S13. System suitability performed on GC-2030 with new met method using standard containing only acetone, ethanol, acetonitrile and triethyl amine.

**GC System ID: CAI2R.EQ.053 (GC-2030)**				
**Injection**	**AUC**<br>**Acetone**	**AUC**<br>**Ethanol**	**AUC**<br>**Acetonitrile**	**AUC**<br>**Triethyl amine**
Injection 1	3210322	75899674	344442	4670673
Injection 2	3202847	75228585	360832	4664982
Injection 3	3180382	75067168	360115	4599950
**Average**	**3197850**	**75398476**	**355130**	**4645202**
**% RSD (≤ 5.0%)**	**0.5**	**0.6**	**2.6**	**0.8**
Confirm system suitability meets requirements.				yes

Table S14. Results from injectable [^{18}F]Fallypride Sample using original ZB624 GC QC Method

**Method: ZB624 GC QC Method**				
**Sample: 18F-FAL-011323-01**				
**Analyte**	**Acetone**	**EtOH**	**MeCN**	**Et_3N**
System Suit. Avg. Counts	2258004	53605304	233762	3270000
Sample Counts	2655	26180624	1214	3886
Sample: S.S. Ratio %	0.00	0.49	0.00	0.00

Table S15. Results from injectable [^{18}F]Fallypride Sample using OMNI Method 2030 QC

**Method: ZB624 OMNI Method 2030 QC**				
**Sample: 18F-FAL-011323-01**				
**Analyte**	**Acetone**	**EtOH**	**MeCN**	**Et_3N**
System Suit. Avg. Counts	3197850	75398476	355130	4645202
Sample Counts	3730	36205510	557	4954
Sample: S.S. Ratio %	0.00	0.48	0.00	0.00

Table S16. Method comparison and cross-validation results for [^{18}F]Fallypride

**Analyte**	**Acetone**	**EtOH**	**MeCN**	**Et_3N**
Sample Ratio % ZB624 GC QC Method	0.00	0.49	0.00	0.00
Sample Ratio % ZB624 OMNI QC Method	0.00	0.48	0.00	0.00
Difference between ratio %	0.00	0.01	0.00	0.00
Difference ≤ 5%?	yes	yes	yes	yes

Table S17. System suitability performed on GC-2014 with original gas chromatography method for [18F]FEPPA using standard containing only methanol, ethanol, and acetonitrile.

**GC System ID: CAI2R.EQ.008 (GC-2014)**			
**Injection**	**AUC Methanol**	**AUC Ethanol**	**AUC Acetonitrile**
Injection 1	987493	39010211	193874
Injection 2	984466	38781331	192287
Injection 3	976407	38453534	183886
**Average**	**982789**	**38748359**	**190016**
**% RSD (≤ 5.0%)**	**0.6**	**0.7**	**2.8**
Confirm system suitability meets requirements.			yes

Table S18. System suitability performed on GC-2030 with new met method using standard containing only acetone, ethanol, acetonitrile and triethyl amine.

**GC System ID: CAI2R.EQ.053 (GC-2030)**			
**Injection**	**AUC Methanol**	**AUC Ethanol**	**AUC Acetonitrile**
Injection 1	1205908	48936745	305568
Injection 2	1208609	49202257	306973
Injection 3	1209254	49043743	304815
**Average**	**1207924**	**49060915**	**305785**
**% RSD (≤ 5.0%)**	**0.1**	**0.3**	**0.4**
Confirm system suitability meets requirements.			yes

Table S19. Results from injectable [18F]FEPPA Sample using original ZB624 FEPPA QC Method

**Method: ZB624 FEPPA QC Method**			
**Sample: 18F-FEPPA-081022-01**			
**Analyte**	**MeOH**	**EtOH**	**MeCN**
System Suit. Avg. Counts	982789	38748359	190016
Sample Counts	46229	33977372	1602
Sample: S.S. Ratio %	4.70	87.69	0.84

Table S20. Results from injectable [18F]FEPPA Sample using OMNI Method 2030 QC

**Method: ZB624 OMNI Method 2030 QC**			
**Sample: 18F-FEPPA-081022-01**			
**Analyte**	**MeOH**	**EtOH**	**MeCN**
System Suit. Avg. Counts	1207924	49060915	305785
Sample Counts	60593	41713992	1792
Sample: S.S. Ratio %	5.02	85.02	0.59

Table S21. Method comparison and cross-validation results for [^{18}F]FEPPA

.

Analyte	MeOH	EtOH	MeCN
Sample Ratio % ZB624 GC QC Method	4.70	87.69	0.84
Sample Ratio % ZB624 OMNI QC Method	5.02	85.02	0.59
Difference between ratio %	0.32	2.67	0.25
Difference ≤ 5%?	yes	yes	yes
